# Evolution‐Driven Bio‐Composite Aerogels for Self‐Adaptive Thermal Regulation and Energy Storage

**DOI:** 10.1002/adma.73935

**Published:** 2026-07-21

**Authors:** Xiaoxue Zhang, Xiaodong Wang, Jianping Zeng, Zhihua Zhang, Shanyu Zhao, Jun Shen

**Affiliations:** ^1^ School of Physics Science and Engineering Tongji University Shanghai China; ^2^ Integrated Systems Laboratory Department of Information Technology and Electrical Engineering ETH Zurich Zurich Switzerland; ^3^ Laboratory for Building Energy Materials and Components Swiss Federal Laboratories for Materials Science and Technology Empa Dübendorf Switzerland

**Keywords:** evolution‐driven aerogel, nature‐inspired multiphase integration, self‐adaptive thermal regulation, universal one‐pot synthesis, zinc‐ion energy storage

## Abstract

Nature achieves remarkable multifunctionality by integrating chemically dissimilar phases into hierarchically organized architectures. Inspired by this principle, we develop a simple and universal strategy to construct bio‐composite aerogels by incorporating trivalent metal chlorides (MCl_3_) into biopolymer chitosan (CTS) matrices. Coordination‐driven assembly in aqueous media enables the direct formation of metal ion‐coordinated chitosan (CTS–M) aerogels without external acids or additional crosslinkers. These aerogels exhibit reversible brittle‐to‐flexible transitions under humidity stimuli, together with exceptional mechanical resilience, enabling self‐adaptive thermal insulation under temperature extremes. Upon pyrolysis, the same precursor is converted into conductive carbon–metal oxide (C–M_2_O_3_) aerogels, where metal oxide nanocrystals are embedded within an interconnected carbon framework. This structural integration couples a continuous electron‐transport network with redox‐active domains, thereby promoting charge‐transfer and increasing accessible storage sites. As a representative example, the C–V_2_O_3_ cathode for aqueous zinc‐ion batteries (ZIBs) delivers excellent energy–power performance (656 Wh kg^−1^ at 200 W kg^−1^, 178 Wh kg^−1^ at ∼20 000 W kg^−1^) with 85% capacity retention after 10 000 cycles, outperforming previously reported carbon–metal oxide systems. By linking adaptive thermal regulation and electrochemical energy storage through a single precursor‐to‐function pathway, this work establishes an evolution‐driven aerogel design paradigm for next‐generation multifunctional materials.

## Graphical abstract

Inspired by nature's multiphase integration, a single chitosan (CTS)–metal chloride (MCl_3_) coordination precursor is engineered to enable sequential material and functional evolution. The resulting metal ion‐coordinated chitosan (CTS–M) aerogels exhibit humidity‐adaptive thermal insulation for battery safety, while carbonization yields conductive carbon–metal oxide (C–M_2_O_3_) aerogels for high‐performance zinc‐ion storage, establishing an evolution‐driven paradigm for multifunctional energy materials.

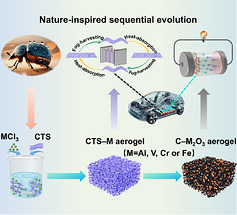

## 1 Introduction

Nature routinely integrates chemically distinct phases into unified, hierarchically organized architectures, enabling multiple, seemingly incompatible functionalities to coexist within a single material system [1]. For instance, in nacre, rigid aragonite platelets are arranged in a brick‐and‐mortar architecture with soft biopolymeric layers, achieving a remarkable balance between strength and toughness [2]. Similarly, bone derives its unique combination of stiffness and damage tolerance from the homogeneous embedding of mineral nanocrystals within a collagen matrix [3]. These biological systems reveal a general design principle: phases with contrasting chemistries can cooperate through controlled hierarchical organization. As a result, functions that are typically mutually exclusive, such as rigidity and flexibility, hydrophilicity and hydrophobicity, or insulation and conductivity, can be integrated within a single material [4, 5, 6, 7, 8, 9, 10].

Aerogels, characterized by ultralow density [11], high porosity [12], and tunable microstructures [13], provide an attractive platform for integrating diverse functional phases within a unified framework [14, 15, 16]. Their open architectures and large internal surface areas are, in principle, well‐suited for accommodating chemically distinct organic and inorganic components. However, achieving a balanced combination of contrasting properties in aerogel systems remains challenging. Organic aerogels derived from polymers or biopolymers offer flexibility and processability but often suffer from limited thermal stability and structural robustness [17, 18], whereas inorganic aerogels, such as silica or metal oxides, exhibit excellent thermal stability and insulation yet are intrinsically brittle and difficult to process [19, 20, 21]. This fundamental incompatibility hinders the development of multifunctional aerogels with coherent phase integration. To address this issue, extensive efforts have focused on organic–inorganic hybrid aerogels via co‐gelation, doping, or gel‐impregnation strategies. Among various candidates, chitosan (CTS) is particularly attractive due to its natural abundance, low toxicity, biocompatibility, and its chemically active amino and hydroxyl groups that facilitate hybridization [22, 23]. Representative studies [24, 25] have demonstrated enhanced mechanical properties or added functionalities; however, these approaches typically involve multistep and energy‐intensive processes, including acid‐assisted dissolution [26], chemical crosslinking [27, 28], prolonged gelation [28], solvent exchange [24], surface modification [24], or supercritical drying [26]. More fundamentally, most existing systems remain structurally static, where the incorporated phases neither undergo continuous evolution nor enable functionally distinct states from a common precursor. This contrasts with biological materials, where multiple phases can emerge sequentially from a single scaffold, giving rise to diverse functionalities. Translating such evolution‐driven multifunctionality into aerogel systems remains largely unexplored yet is crucial for advancing next‐generation multi‐role materials. Therefore, developing a simple, scalable, and chemically general strategy that enables coherent phase integration alongside controlled compositional evolution and functional diversification is highly desirable.

Here, inspired by nature's principle of multiphase integration and functional divergence, we develop a unified synthetic platform based on a molecularly integrated CTS–metal chloride (MCl_3_) coordination system. Distinct from conventional CTS processing that relies on external acids, this approach enables direct dissolution, coordination assembly, and aerogel formation in a one‐pot manner using only CTS and MCl_3_ (with water as the solvent). The hydrolysis‐induced acidity of MCl_3_ simultaneously drives polymer dissolution and coordination network formation, yielding a homogeneous precursor that integrates organic and inorganic components at the molecular level. Notably, this system features an unusually simplified yet highly integrated materials design, in which each component performs multiple indispensable roles throughout the entire process. MCl_3_ acts as the dissolution medium, coordination node, hygroscopic phase, and metal precursor, while CTS serves as the structural scaffold, moisture‐storage framework, and carbon source. As a result, all functionalities emerge from the intrinsic role multiplexing of these two constituents, enabling a zero‐redundancy precursor system with continuous functional utilization. Building on this chemically unified and functionally integrated precursor, we further establish an evolution‐driven material pathway in which structure and properties emerge through staged transformation rather than independent design. In the first stage, humidity‐responsive CTS–M aerogels exhibit reversible moisture‐triggered brittle‐to‐flexible transitions, enabling adaptive thermal–mechanical regulation. In the second stage, high‐temperature pyrolysis converts the same precursor into C–M_2_O_3_ hybrid aerogels, where in situ formed metal oxide nanocrystals are confined within a conductive carbon network to facilitate efficient charge transport and high electrochemical activity. As a representative example, the Zn//2 M ZnSO_4_//C–V_2_O_3_ ZIB delivers a high specific capacity of 341 mAh g^−1^ at 0.1 A g^−1^ and achieves 85% capacity retention after 10 000 cycles. By bridging humidity‐adaptive thermal management and electrochemical energy storage within a single precursor‐to‐function continuum, this work establishes a generalizable paradigm in which multifunctionality arises from controlled material evolution rather than additive integration, offering a scalable pathway toward next‐generation self‐adaptive aerogels for energy, electronics, and safety‐critical applications.

## 2 Results and Discussion

### 2.1 Nature‐Inspired Precursor Design and Staged Evolution

#### 2.1.1 Coordination‐Driven Self‐Assembly of CTS–M Aerogels

**FIGURE 1:**
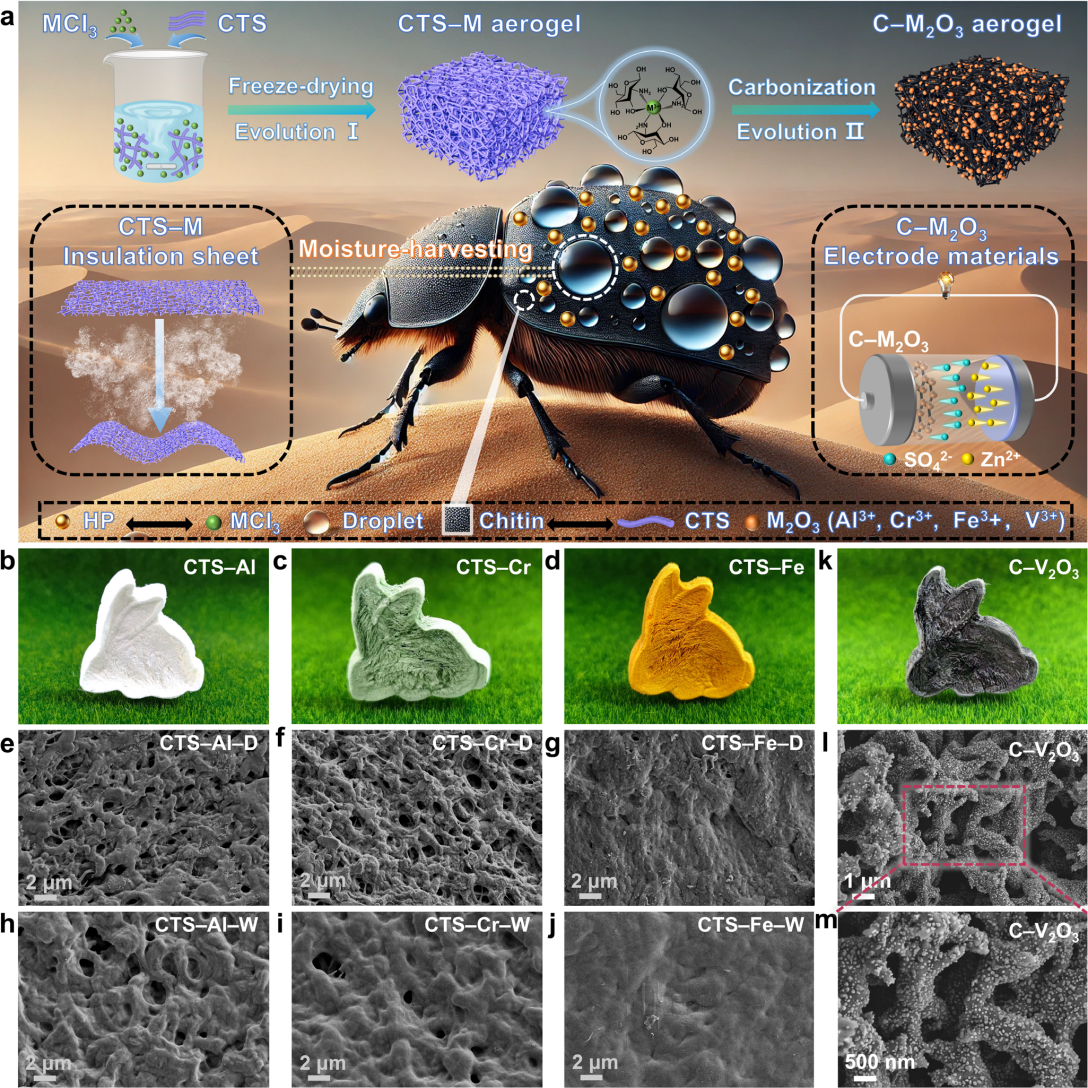
Nature‐inspired design and structural evolution of CTS–M and C–M_2_O_3_ aerogels. (a) Schematic illustration of the nature‐inspired design concept and the two‐stage evolutionary pathway from CTS–MCl_3_ precursors to C–M_2_O_3_ aerogels. Hygroscopic MCl_3_ mimics the hydrophilic protuberances (HPs) of the beetle, while the CTS framework represents the chitinous exoskeleton. Photographs of representative CTS–Al (b), CTS–Cr (c), and CTS–Fe (d) aerogels. SEM images of CTS–M aerogels before (e–g) and after (h–j) moisture sorption (D = dry, W = moisture‐conditioned). Photograph (k) and SEM images (l,m) of C–V_2_O_3_ aerogels (derived from tan CTS–V precursor).

Figure 1a illustrates the design inspiration and synthesis of CTS–M and C–M_2_O_3_ aerogels, inspired by the fog‐harvesting mechanism of the Namib Desert beetle. The beetle's exoskeleton contains hydrophilic protuberances (HPs) that capture airborne moisture, which subsequently condenses and is transported along hydrophobic regions. Analogously, hygroscopic M species serve as moisture‐capturing sites, while CTS, a biopolymer, provides the structural framework. CTS–M aerogels are fabricated via a one‐pot mixing and freeze‐drying process, followed by high‐temperature pyrolysis under nitrogen atmosphere to yield C–M_2_O_3_ composite aerogels through in situ growth of metal oxide nanocrystals within a carbonized CTS skeleton (Experimental details in Supporting Information). This strategy relies only on CTS, MCl_3_, and water, eliminating the need for external acids (e.g., acetic or hydrochloric acid) typically required for CTS dissolution. The direct dissolution of CTS in hydrolyzed MCl_3_ solutions simplifies the process and improves scalability. The inset in Figure 1a depicts the coordination interaction between trivalent metal ions and the amino/hydroxyl groups of CTS, forming octahedral 6‐coordinated complexes. This coordination is supported by Fourier transform infrared (FTIR) and x‐ray diffraction (XRD) analyses (Figures S1 and S2). In FTIR spectra, the O─H/N─H stretching (∼3441 cm^−1^) [29] and N─H bending (∼1641 cm^−1^) [30] bands in all CTS–M aerogels exhibit redshifts relative to pristine CTS aerogel, indicating coordination with ─OH and ─NH_2_ groups [23]. The secondary hydroxyl peak (∼1074 cm^−1^) shows reduced intensity and a slight blueshift, further confirming this interaction. XRD patterns display broad amorphous features without crystalline CTS or MCl_3_·6H_2_O peaks, suggesting that coordination interactions between trivalent metal ions and lone‐pair electrons of amino and hydroxyl groups compete with and partially disrupt the native hydrogen‐bonding network of CTS, thereby enabling homogeneous incorporation of metal ions into the polymer matrix [31, 32]. As a result, CTS–M aerogels retain the intrinsic thermal insulation of CTS aerogels while exhibiting a humidity‐triggered brittle‐to‐flexible transition, making them suitable for adaptive thermal regulation. After carbonization, the resulting C–M_2_O_3_ aerogels integrate the high electrical conductivity of carbon networks with the high theoretical capacities of metal oxides, enabling enhanced charge storage and ion/electron transport for ZIB applications.

Figure 1b–d shows CTS–M aerogels sculpted into rabbit‐like shapes, demonstrating excellent machinability. The CTS–Al, CTS–Cr, and CTS–Fe aerogels exhibit distinct colors (white, green, and orange) and ultralow densities of 0.068, 0.071, and 0.073 g cm^−3^ (Table S1), respectively. Their lightweight nature is illustrated in Figure S3, where a CTS–Fe aerogel rests on a flower without deforming it. Macroscopically, the aerogels display a rough texture with directional fibrous features originating from ice templating during freeze‐drying, along with visible wrinkles and interconnected macropores favorable for moisture sorption. Scanning electron microscopy (SEM) images (Figure 1e–j and Figures S4 and S5) reveal a three‐dimensional (3D) porous network composed of macropores and fibrous skeletons with smooth pore walls and no evident aggregation in all CTS–M aerogels. Energy‐dispersive X‐ray spectroscopy (EDS) mapping of CTS–Cr (Figure S5b) confirms homogeneous distribution of metal ions (e.g., Cr^3^^+^) within the CTS framework, providing uniformly distributed hygroscopic sites. Upon exposure to ambient humidity, CTS–M aerogels undergo pronounced morphological changes (Figure 1h−j and Figure S4e−h), including pore reduction, fibrous skeleton swelling, and overall densification, reflecting moisture‐induced structural rearrangement. The appearance of localized lamellar wrinkles (highlighted in Figure S4e–g) further suggests structural softening that is consistent with the humidity‐responsive mechanical behavior discussed below.

#### 2.1.2 Carbonization‐Driven Evolution From CTS–M to C–M_2_O_3_ Aerogels

**FIGURE 2:**
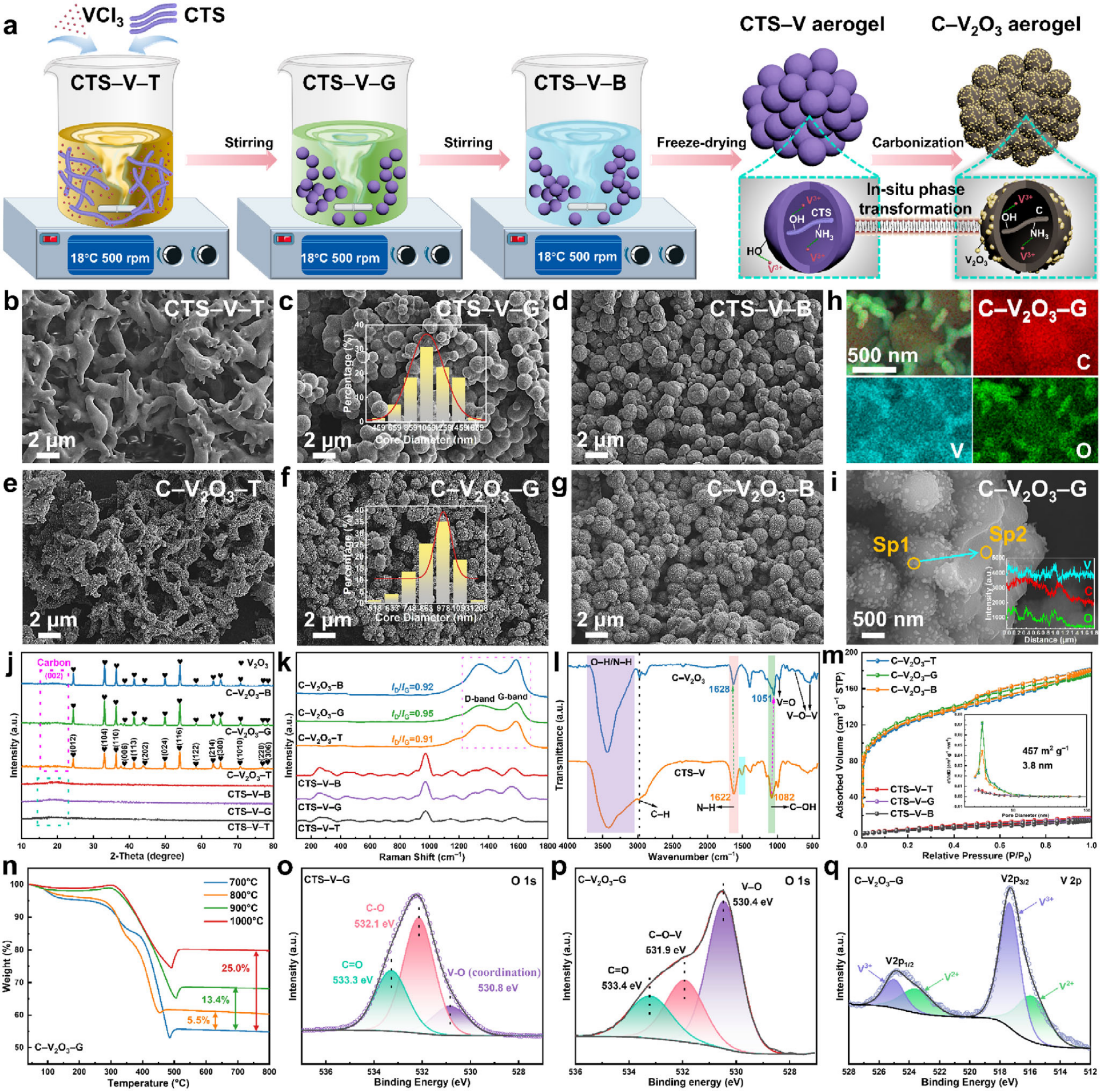
Formation and characterization of C–V_2_O_3_ aerogels. (a) Schematic illustration of the synthesis process for C–V_2_O_3_ aerogels. CTS–V–T, CTS–V–G, and CTS–V–B correspond to tan, green, and blue precursor samples, respectively; their carbonized counterparts are denoted as C–V_2_O_3_–T, CTS–V–G, and CTS–V–B. SEM images of CTS–V–T (b), CTS–V–G (c), and CTS–V–B (d) aerogels; the inset in (c) shows the particle size distribution. SEM images of C–V_2_O_3_–T (e), C–V_2_O_3_–G (f), and C–V_2_O_3_–B (g) aerogels; inset in (f) shows the particle size distribution. (h) EDS elemental mapping of C, V, and O in C–V_2_O_3_–G aerogel. (i) SEM line‐scan image of C–V_2_O_3_–G aerogel (arrow indicates the scan trajectory); the inset shows the corresponding element distribution. XRD patterns (j), Raman spectra (k), FTIR spectra (l), and N_2_ adsorption–desorption isotherms (m) of samples before and after carbonization; the inset in (m) shows pore size distribution. (n) TGA curves of C–V_2_O_3_–G aerogels under an air atmosphere. High‐resolution O 1s XPS spectra of CTS–V (o) and C–V_2_O_3_ (p) aerogels. (q) High‐resolution V 2p XPS spectrum of C–V_2_O_3_ aerogel.

Building upon the CTS–M aerogel architecture, carbonization drives the precursors into a second evolution stage, generating carbon/metal‐oxide hybrid aerogels (Figures S6−S8). As exemplified by C–V_2_O_3_, the CTS–V aerogel was transformed into a C–V_2_O_3_ hybrid aerogel via an in situ confined‐growth process, enabling the integration of high electrical conductivity with large charge‐storage capacity (Figure 2a). During sol formation, the CTS–V solution underwent a distinct color evolution from tan to green and finally to blue (Figure 2a), reflecting progressive changes in the coordination environment and electronic state of V^3+^ ions. This molecular‐level coordination modulated the self‐assembly pathway of CTS, driving a morphological transition from fibrous structures (CTS–V–T) to spherical aggregates (CTS–V–G and CTS–V–B), as shown in Figure 2b–d. This transformation suggests that multipoint chelation of V^3+^ disrupts the native hydrogen‐bonding network of CTS, thereby triggering structural reorganization. EDS elemental mapping (Figure S9d,h,l) confirms the homogeneous distribution of C, O, and V throughout the framework, verifying uniform molecular‐scale incorporation of V^3+^ within the CTS network. After carbonization, the resulting C–V_2_O_3_ aerogel retains its monolithic form (Figure 1k), preserving the macroscopic integrity and processability of the CTS–V aerogel. The interconnected 3D porous skeleton is largely maintained, while pyrolysis induces the formation of nanoparticle‐decorated surfaces (Figure 2e–g), yielding a distinct core‐shell structure (Figure 2f,g). Higher‐magnification SEM images (Figure 1l,m) further confirm the uniform spatial distribution of these nanoscale particles. Particle size analysis (insets of Figure 2c,f) reveals a reduction in average sphere diameter from 1059 to 978 nm after carbonization, attributed to skeleton shrinkage caused by thermal decomposition and volatilization of organics, resulting in a denser and more stable microstructure. Elemental mapping and line‐scan profiles (Figure 2h,i) show enrichment of V and O at the particle surfaces, with an amorphous carbon matrix in the interior. High‐resolution Transmission electron microscope (TEM) images (Figure S10) further identify lattice fringes corresponding to the (104), (110), (012), and (113) planes of crystalline V_2_O_3_ [33], interspersed with disordered carbon domains. These observations confirm the formation of a dual‐phase structure consisting of surface‐anchored V_2_O_3_ nanocrystals and vanadium species confined within the carbon skeleton. This structural evolution is further supported by spectroscopic and diffraction analyses. XRD patterns (Figure 2j) of CTS–V aerogels show only a broad amorphous halo at 15°−23°, whereas carbonized C–V_2_O_3_ aerogels exhibit sharp diffraction peaks indexed to crystalline V_2_O_3_ (JCPDS No. 34–0187). A weak diffuse peak at ∼20° (Figure S11b) further indicates the presence of disordered carbon (002) [34]. Thermogravimetric analysis (TGA) (Figure 2n and Figures S12−S14) shows that the carbon content decreases with increasing carbonization temperature, accompanied by a corresponding increase in the V_2_O_3_ fraction. Raman spectra (Figure 2k) display prominent D (∼1346cm^−1^) and G (∼1586 cm^−1^) bands (*I*_D_/*I*_G_ = 0.91−0.95) in C–V_2_O_3_ aerogels, indicating partial graphitization of the carbon framework. Point‐resolved Raman spectra (Figure S15) show strong D and G bands in the inner core regions (Sp^1^−Sp^3^), while the outer shell regions (Sp^4^ and Sp^5^) exhibit only V_2_O_3_‐related signals, confirming a carbon‐core/V_2_O_3_‐shell hybrid structure. FTIR spectra (Figure 2l) identify C─O stretching of the CTS backbone (1082 cm^−1^) [35] and N─H bending (1622 cm^−1^) vibrations as the primary coordination sites for V^3+^ prior to carbonization. After carbonization, these peaks shift, and new V═O (974 cm^−1^) [36] and V─O─V (748 and 524 cm^−1^) [37] vibrations emerge, indicating partial ligand dissociation and in situ formation of V_2_O_3_ nanocrystals via oxygen release from coordinating groups [36, 37]. This transformation occurs under an inert atmosphere, highlighting ligand‐derived oxygen as the internal oxidant and eliminating the need for external oxygen sources. These results demonstrate the successful transformation of an amorphous coordination polymer into crystalline V_2_O_3_ embedded within a conductive carbon matrix, thereby enabling enhanced electrical conductivity and electrochemical performance.

Beyond phase transformation, carbonization also tailors the porosity and charge transport properties of the aerogels. As shown in Figure 2m, C–V_2_O_3_ aerogels exhibit typical type IV nitrogen adsorption–desorption isotherms with pronounced H3 hysteresis loops, suggesting slit‐shaped mesopores formed by agglomerates of plate‐like nanostructures [38]. The pore‐size distribution (inset) is centered at ∼3.8 nm, further confirming the mesoporous nature. Carbonization significantly increases the specific surface area (SSA) from 20 to 457 m^2^ g^−1^ and enlarges the pore volume by nearly an order of magnitude (Table S2). X‐ray photoelectron spectroscopy (XPS) survey spectra (Figure S16) confirm the presence of C, O, V, and N in both CTS–VCl_3_ precursors of different colors and the corresponding C–V_2_O_3_ aerogels. The absence of Cl signals after carbonization indicates effective removal of precursor residues and improved material purity. The O 1s spectra (Figure 2o, p) evolve from V─O coordination in CTS–V aerogels to crystalline V─O and interfacial C─O─V bonds in C–V_2_O_3_ aerogels [39], reflecting the transition from molecular‐level chelation to metal oxide formation. The V 2p spectrum (Figure 2q) reveals coexisting V^3+^ and V^2+^ species, suggesting the presence of oxygen vacancies in V_2_O_3_ domains that can enhance electrochemical activity [40]. The N 1s spectra (Figure S17d,h) reveal a clear evolution of nitrogen species during carbonization. The amino and protonated/coordinated nitrogen species in the CTS‐VCl_3_ precursors are converted into pyridinic‐N and graphitic‐N within the carbon framework after pyrolysis. The resulting graphitic‐N improves electronic conductivity, whereas pyridinic‐N provides additional active sites for interfacial charge‐transfer reactions [41]. Notably, similar spectral evolutions are observed for all precursor‐derived samples with different initial colors (Figures S17−S19), indicating that these carbonization‐induced chemical transformations are independent of precursor color. Furthermore, valence band spectra (Figure S20) display sharper band edges and increased density of states near the Fermi level after carbonization, suggesting enhanced electronic delocalization and improved charge transport. These changes demonstrate that carbonization endows the aerogels with hierarchical porosity and optimized electronic structure, both of which are beneficial for high‐performance energy storage.

### 2.2 Evolution‐Driven Functional Divergence

#### 2.2.1 Self‐Adaptive Thermal Regulation in CTS–M Aerogels

**FIGURE 3:**
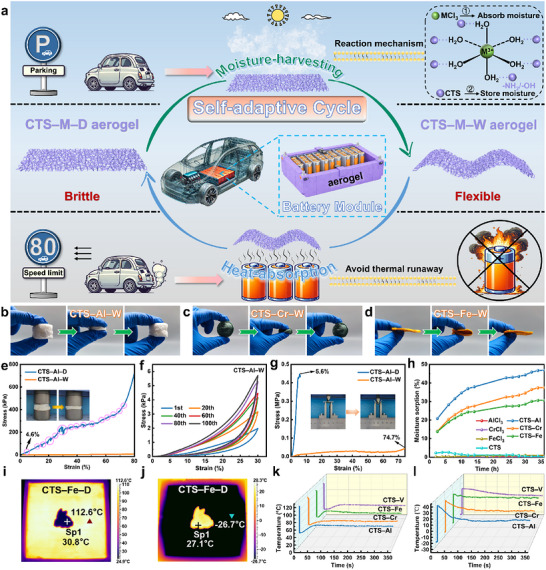
Self‐adaptive mechanical response and thermal insulation performance of CTS–M aerogels. (a) Schematic of the humidity–temperature self‐adaptive cycle of CTS–M aerogel as an external thermal buffer/protective layer for electric vehicle battery modules. Photographs of hydrated CTS–Al (b), CTS–Cr (c), and CTS–Fe (d) aerogels under finger compression and bending. (e) Uniaxial compression stress–strain curves of CTS–Al aerogel before and after moisture sorption, with corresponding photographs. (f) Compression–release curves of hydrated CTS–Al aerogel at 30% strain after moisture sorption–evaporation cycles. (g) Three‐point bending stress–strain curves of CTS–Al aerogel before and after moisture sorption, with corresponding photographs. (h) Moisture sorption behavior of CTS aerogel, MCl_3_ salts, and CTS–M aerogels under dynamically varying ambient humidity. Infrared thermal images of CTS–Fe–D aerogel placed on a 110°C hot plate (i) and a −196°C cold plate (j). (*Note*: the apparent temperature of the cold plate is limited by the detection range of the infrared thermal imager.) Time‐dependent rear‐surface temperature profiles of CTS–M aerogels on a hot plate (k) and a cold plate (l).

CTS–M aerogels exhibit a unique synergy of humidity‐responsive behavior and adaptive thermal regulation. Figure 3 illustrates this concept through a reversible working cycle (Figure 3a), in which mechanical compliance and thermal insulation are dynamically regulated via humidity‐triggered moisture sorption and heat‐induced desorption. Under stationary conditions, when batteries are not generating heat, CTS–M aerogels capture ambient moisture through M^3+^‐ion‐mediated hydration. The absorbed water is stabilized by hydrogen bonding with the CTS backbone and confined within the hierarchical pore network, leading to effective plasticization of the aerogel and a transition from a brittle to a flexible state. This mechanical compliance enables conformal contact and provides buffering against mechanical disturbances, vibrations, and assembly stresses during parking or routine operation. During battery operation, heat generation drives moisture desorption, restoring the aerogel to a dry and mechanically stiffened state that favors thermal insulation. In addition, dehydration‐induced contraction of the CTS–M aerogels may provide structural tolerance to accommodate volume expansion of battery modules during charge–discharge cycles. In practical configurations, these aerogels can be integrated as thermal‐buffering or protective interlayers surrounding battery modules, where adaptive thermal insulation and mechanical buffering are simultaneously achieved. Within such architectures, the sorbed moisture remains confined within the porous framework and participates in a dynamic sorption–evaporation equilibrium, enabling reversible thermal–mechanical regulation under fluctuating operating conditions. The measured moisture sorption kinetics, humidity‐induced mechanical transition, and thermal insulation performance (Figure 3b−l and Figures S21−S49) collectively support the feasibility of this adaptive material concept.

To approximate practical operating conditions, moisture‐sorption tests were conducted under an indoor environment with open windows, where temperature and humidity fluctuate dynamically. Under these conditions, CTS–Al, CTS–Cr, and CTS–Fe aerogels reach equilibrium uptakes of 44.3%, 34.5%, and 28.6%, respectively—significantly higher than those of pristine CTS aerogel or MCl_3_ salts (<2.7%, Figure 3h). Among them, CTS–Al aerogel exhibits both the highest uptake and fastest sorption kinetics (initial slope), which can be attributed to the stronger hydration tendency of Al^3+^ compared to Cr^3+^ and Fe^3+^ ions [42]. Notably, pristine CTS aerogel shows an initial increase followed by a slight decrease in moisture uptake (Figure 3h and Figure S21), which arises from redistribution and desorption of weakly bound water, a behavior commonly observed in hygroscopic gels [43]. In contrast, CTS–M aerogels display stable and monotonic uptake, owing to the combined effect of strong metal cation hydration and an interconnected porous framework that facilitates water transport and retention. Direct observations (Figure S22) further highlight this cooperative mechanism: MCl_3_ salts undergo severe deliquescence (Figure S22a), while pristine CTS aerogel shows negligible morphological change (Figure S22b); in comparison, hydrated CTS–M aerogels retain structural integrity without deliquescence (Figure S22c,d). This indicates that M^3+^ ions function as active hygroscopic centers driving water uptake, while the CTS matrix acts as a stabilizing scaffold that preserves the aerogel structure and enables uniform water distribution and storage. FTIR analysis (Figure S23) supports this interpretation, showing intensified O─H and N─H stretching bands (normalized to the C─H peak at ∼2974 cm^−1^), indicative of strengthened hydrogen bonding, while the characteristic CTS peaks remain unchanged, confirming chemical stability. Additional time‐dependent ambient and controlled‐humidity experiments reveal that the brittle‐to‐flexible transition is threshold‐dependent rather than instantaneous (Figures S24−S28). At low moisture uptake, only partial softening in CTS–M aerogel is observed, accompanied by discontinuous stress drops from localized fracture, whereas a fully flexible response emerges once the absorbed moisture exceeds a critical range (∼0.21–0.26 g g^−1^, depending on the metal species). These results suggest that the mechanical state is governed by the internal moisture content, which is in turn regulated by humidity and exposure time. Furthermore, sorption–evaporation cycling tests under both dynamic ambient conditions and controlled relative humidity (RH ≈ 68%) demonstrate good reversibility, with each cycle maintaining sufficient moisture uptake to sustain the brittle‐to‐flexible transition (Figure S29).

Figure 3b–g illustrates the moisture‐induced mechanical softening of CTS–M aerogels. Finger compression and bending tests (Figure 3b−d and Movie S1) clearly show a transition from brittle to flexible behavior upon moisture uptake. Under uniaxial compression (Figure 3e), the dry CTS–Al aerogel exhibits initial brittle failure at ∼4.6% strain with characteristic stepwise stress drops. In contrast, the hydrated sample undergoes smooth deformation up to 80% strain without fracture, indicating a moisture‐induced shift to a flexible regime. Compression tests after repeated moisture sorption–evaporation cycles (Figure 3f) further demonstrate the stability of this behavior, with the hydrated CTS–Al aerogel maintaining a consistent response over 100 cycles at 30% strain. A similar trend is observed in three‐point bending tests (Figure 3g): the dry CTS–Al aerogel fractures abruptly at ∼5.6% bending strain, whereas the hydrated aerogel tolerates progressive deformation up to 74.7% without visible cracking (insets), confirming a humidity‐induced enhancement in mechanical compliance. Comparable responses are observed for CTS–Cr and CTS–Fe aerogels (Figure S30), as also evidenced in Movie S1.

To quantitatively evaluate this adaptive mechanical transition under thermally relevant conditions, temperature‐dependent compression tests were performed on moisture‐conditioned CTS–M aerogels (Figures S31–S33). The compressive modulus increases progressively with temperature, reflecting gradual stiffening as retained moisture decreases upon heating. Taking CTS–Fe–W as a representative example, the modulus increases from 0.16 kPa at 20°C to 0.39, 0.56, and 1.24 kPa at 40°C, 60°C, and 80°C, respectively (Figure S33). Among the studied systems, CTS–Al–W maintains the highest compliance at elevated temperature (Figure S31c,d), consistent with its higher moisture retention (Figure S32). These results establish a clear modulus–temperature relationship, confirming that the mechanical transition is governed by the coupled effects of moisture retention and thermally induced desorption. Within the investigated humidity range, moisture uptake remains self‐limited even at high humidity (Figure S34), and the hydrated aerogels exhibit fully recoverable mechanical behavior upon cycling (Figure 3f), indicating that the brittle‐to‐flexible transition is reversible and does not involve significant structural degradation. Importantly, no visible wet traces were observed when hydrated CTS–M aerogels were compressed on dry paper (Figure S35 and Movie S2), demonstrating that the absorbed water is confined within the porous network rather than existing as free liquid.

To further elucidate the origin of this moisture‐induced softening, a unified structure‐property correlation was established (Figures S23 and S36–S47 and Tables S3 and S4). SAXS 2D patterns (Figure S36) display concentric scattering rings without diffraction features, confirming the amorphous nature of both dry and hydrated aerogels [44, 45]. Upon moisture uptake, several characteristic changes are observed, including blurred scattering rings (Figure S36d–f), reduced scattering intensity (Figure S37a), slope variations in both low‐ and high‐q regions (Figure S37b), and flattened Kratky profiles (Figure S38). These features indicate partial pore filling and pore‐wall swelling, consistent with hydration‐induced structural rearrangements reported in biopolymer aerogels [46]. Such evolution reduces electron‐density contrast between the solid framework and pores, weakens inter‐chain interactions, and induces local network relaxation, thereby providing a structural basis for the observed reversible softening [47, 48, 49]. Further evidence is provided by weakened Porod deviations (Figure S39), reduced fractal dimensions (e.g., CTS–Cr: 2.7 to 2.4; CTS–Fe: 2.5 to 2.3; Figure S40), and narrowed PDDF peaks (Figure S41), all of which indicate water infiltration‐induced structural homogenization [46, 50]. These findings are consistent with SEM observations showing pore filling, wall thickening, and surface smoothing (Figure 1h−j). Representative pore structure analysis of CTS–Al further supports this mechanism, as evidenced by a pronounced decrease in surface area (9.7 to 0.5 m^2^ g^−1^) and pore volume (0.020 to 0.001 cm^3^ g^−1^; Figure S42 and Table S3). To differentiate possible chemical or phase changes, XRD, XPS, and FTIR analyses were performed. XRD patterns (Figure S43) show no new diffraction peaks, confirming the absence of phase transformation. XPS spectra (Figures S44–S47) display stable metal oxidation states, indicating that the metal−hydroxyl/amino coordination framework is largely retained after moisture sorption. FTIR spectra (Figure S23) exhibit intensified O─H/N─H stretching bands (normalized to the C─H peak at ∼2974 cm^−1^), consistent with hydrogen‐bond reorganization without backbone degradation. Collectively, these findings demonstrate that moisture uptake induces reversible pore‐network relaxation and structural homogenization without significant chemical transformation, thereby alleviating stress concentration and enabling flexible deformation [46, 48].

Thermal insulation performance under extreme conditions is demonstrated in Figure 3i–l. When placed on a 110 °C hot plate and a −196°C cold plate, CTS–Fe aerogel maintains surface temperatures of 30.8°C and 27.1°C, respectively (Figure 3i,j), indicating effective insulation under both high and low temperatures. Similar behavior is observed for other CTS–M aerogels (Figures S48 and S49), where infrared imaging reveals pronounced vertical temperature gradients, directly visualizing their insulation capability. Time‐dependent rear‐surface temperature profiles (Figure 3k, l) show that temperatures remain below 60°C (hot plate) and above 9°C (cold plate) over 6 min, providing a meaningful thermal buffering window for safety‐critical scenarios. These results demonstrate that CTS–M aerogels combine humidity‐responsive mechanical adaptability with thermal insulation, supporting their potential as self‐adaptive protective materials for battery systems.

#### 2.2.2 High‐Performance Zinc‐Ion Storage in C–M_2_O_3_ Aerogels

**FIGURE 4:**
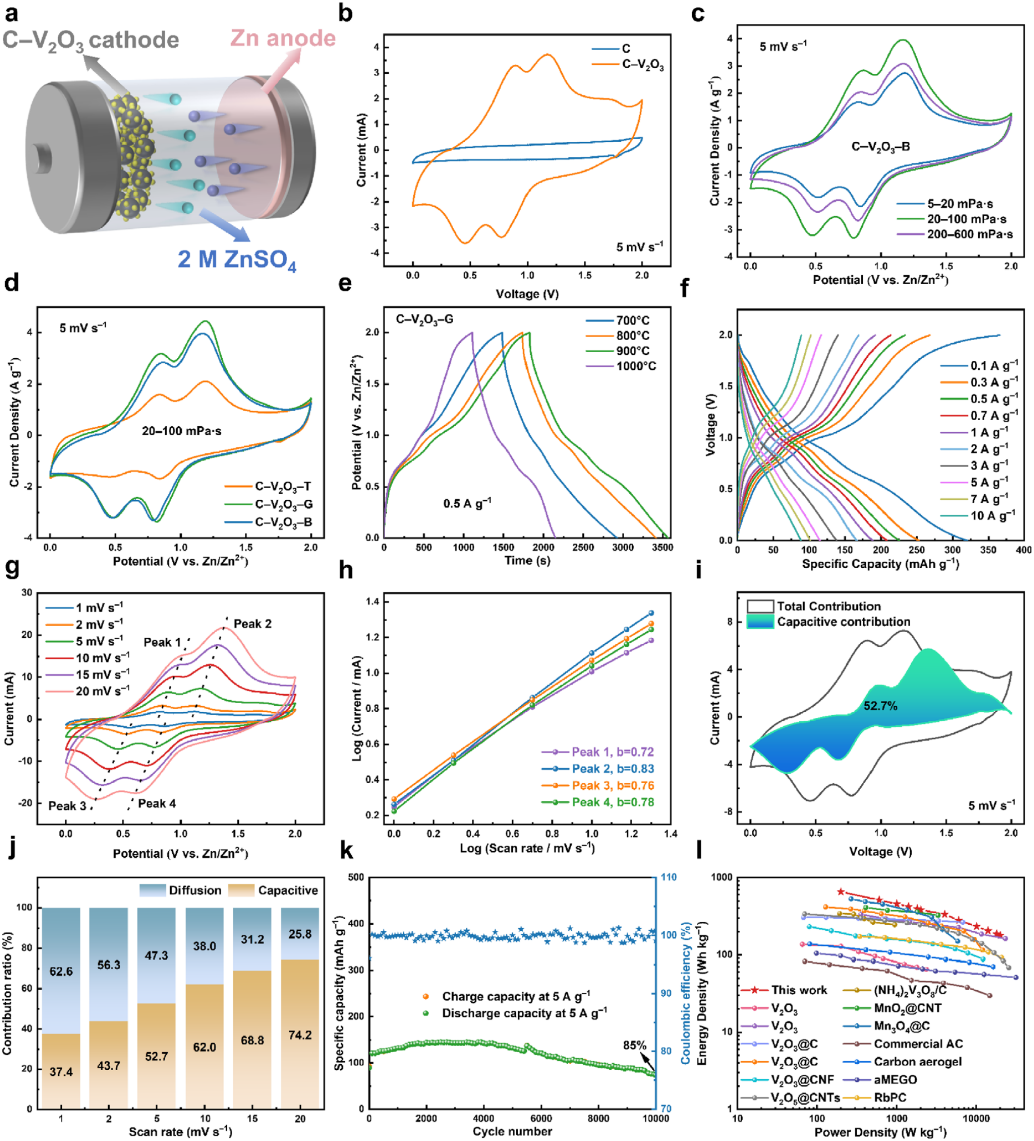
Electrochemical performance of C–V_2_O_3_ aerogel electrode in aqueous ZIBs. (a) Schematic illustration of the aqueous Zn//2 M ZnSO_4_//C–V_2_O_3_ ZIB configuration. (b) Comparison of CV curves of carbon and C–V_2_O_3_ aerogels at 5 mV s^−1^. CV curves of C–V_2_O_3_ aerogels derived from CTS–VCl_3_ precursors with different viscosities (c) and colors (d) at 5 mV s^−1^. (e) GCD curves of C–V_2_O_3_–G aerogels carbonized at different temperatures (700°C−1000°C) measured at 0.5 A g^−^^1^. Electrochemical performance of the optimized C–V_2_O_3_–G–900 electrode: (f) GCD curves at different current densities; (g) CV curves at different scan rates; (h) log(current) versus log(scan rate) plots at the peak current; (i) capacitive contribution (blue‐shaded areas) at 5 mV s^−1^; (j) capacitive and diffusion‐controlled contributions at various scan rates; (k) cycling performance and Coulombic efficiency; (l) Ragone plots comparison.

Upon carbonization, the system enters a second evolution stage, yielding conductive C–M_2_O_3_ aerogels optimized for electrochemical energy storage. To evaluate their performance, aqueous Zn//2 M ZnSO_4_//C–V_2_O_3_ ZIBs were assembled (Figure 4a). As shown in Figure 4b, the carbon (C) electrode derived from acetic‐acid‐dissolved CTS exhibits a quasi‐rectangular cyclic voltammetry (CV) profile, characteristic of electric double‐layer capacitance. In contrast, the C–V_2_O_3_ electrode prepared via the VCl_3_‐dissolved route displays two pairs of well‐defined redox peaks together with a significantly enlarged CV area, indicating the coexistence of capacitive charge storage from the carbon framework and diffusion‐controlled Faradaic reactions from V_2_O_3_. Consistent behavior is observed in galvanostatic charge–discharge (GCD) measurements (Figure S50), where the C–V_2_O_3_ electrode presents two discharge plateaus and extended discharge time. The influence of precursor parameters was further examined (Figure 4c−e). All samples show two pairs of redox peaks (1.19/0.82 and 0.85/0.49 V), corresponding to reversible Zn^2+^ insertion/extraction. Among different viscosities (Figure 4c), the medium‐viscosity CTS (20−100 mPa·s) yields the largest CV area and the most symmetric redox peaks, suggesting improved electrochemical reversibility. This trend is corroborated by GCD results (Figure S51). Structurally, this sample exhibits a uniform and densely packed microspherical morphology, whereas low‐viscosity precursors lead to loose structures and high‐viscosity systems produce aggregated morphologies (Figure S52). Raman analysis (Figure S53a) further shows a relatively high 𝐼_D_/*I*_G_ ratio (0.95), indicating a defect‐rich carbon structure that facilitates ion/electron transport. A similar dependence is observed for precursors with different colors (Figure 4d), where the C–V_2_O_3_–G sample shows the largest CV area and the longest discharge time (Figure S54a). Electrochemical impedance spectroscopy (EIS, Figure S54b) reveals the lowest charge‐transfer resistance (*R*_ct_) for C–V_2_O_3_–G, while a small contact angle (20.1°, Figure S55) indicates improved electrolyte wettability. Combined structural analyses (Figures S56, S57a and Table S2) suggest that this performance is associated with its uniform mesoporous spherical structure, relatively high crystallinity, and large SSA (457 m^2^ g^−1^). Optimization of carbonization temperature (Figure 4e and Figure S58) identifies 900°C as the most favorable condition. At this temperature, the electrode exhibits the highest capacity, more symmetric CV/GCD profiles, and the lowest *R*_ct_. XRD patterns (Figure S57b) show well‐defined V_2_O_3_ peaks and weak VN features, suggesting a possible V_2_O_3_–VN synergy that may enhance electrical conductivity. TGA analysis (Figure S14) indicates a balanced C/V_2_O_3_ mass ratio of 0.53 in C–V_2_O_3_ aerogels carbonized at 900°C, while Raman spectra (Figure S53b) show a relatively high 𝐼_D_/*I*_G_ ratio (0.96), reflecting a defect‐enriched yet partially graphitized carbon framework. By comparison, lower carbonization temperatures (700°C−800°C) lead to insufficient crystallinity and structural development (Figures S53b, S57b and S59), whereas excessive temperature (1000°C) increases graphitization but reduces defect density. The 900°C condition therefore provides a balanced defect–order structure, facilitating both ion storage and charge transport [51, 52].

The rate performance of the optimized C–V_2_O_3_–G–900 electrode was evaluated using GCD measurements from 0.1 to 10 A g^−1^ (Figure 4f). Two distinct discharge plateaus are retained across current densities, indicating reversible redox reactions. The electrode delivers a specific capacity of 341 mAh g^−1^ at 0.1 A g^−1^ and maintains 89 mAh g^−1^ at 10 A g^−1^ (Figure S60), demonstrating good rate capability. CV analysis at varying scan rates (Figure 4g) reveals broadened and shifted redox peaks. The *b*‐values derived from the power‐law relationship *i* = a*v^b^* (Figure 4h) range from 0.72 to 0.83, indicating combined capacitive and diffusion‐controlled contributions. Quantitative analysis (*i*(*V*) = *k*_1_ *v*+*k*_2_ *v* ^1/2^) shows that the capacitive contribution increases with scan rate (Figure 4j), reaching 74.2% at 20 mV s^−1^ (Figure 4i and Figure S61), consistent with enhanced surface‐controlled kinetics. To further correlate the characteristic structure with electrochemical behavior, ex situ XRD and Raman measurements were performed at representative charge/discharge states (Figures S62 and S63). The V_2_O_3_ phase preserves its characteristic diffraction peaks, accompanied by reversible peak shifts during Zn^2+^ insertion/extraction, while the carbon framework retains stable D and G bands with only minor variation in *I*_D_/*I*_G_ ratio (0.97−1.06). These results indicate that the excellent electrochemical performance arises from the combination of a structurally stable conductive network and a redox‐active oxide phase capable of reversible structural evolution during Zn^2^^+^ insertion/extraction.

Cycling stability was evaluated at 5 A g^−^^1^ (Figure 4k and Figure S64). After an initial activation stage, the capacity gradually stabilizes, with 85% capacity retention after 10 000 cycles and 50% after 120 000 cycles. The Coulombic efficiency remains close to 100% throughout, indicating high reversibility. Post‐cycling SEM images (Figure S65) show that the overall spherical morphology is largely preserved, with only minor particle size reduction and interface relaxation. In contrast, the Zn anode exhibits surface roughening and flake‐like byproduct deposition, suggesting that capacity decay is primarily associated with anode degradation rather than cathode instability. The Ragone plot (Figure 4l) demonstrates energy densities of 656 Wh kg^−1^ at 200 W kg^−1^ and 178 Wh kg^−1^ at ∼20,000 W kg^−1^, comparing favorably with previously reported pristine V_2_O_3_ [53, 54], carbon‐based materials [55, 56, 57, 58], V_2_O_3_/carbon composites [59, 60, 61], and other metal oxide/carbon composites [62, 63, 64, 65]. In addition to energy−power characteristics, comparison with representative aqueous ZIB cathodes (Table S5) highlights the coexistence of relatively high specific capacity and long cycling stability in this system.

**FIGURE 5:**
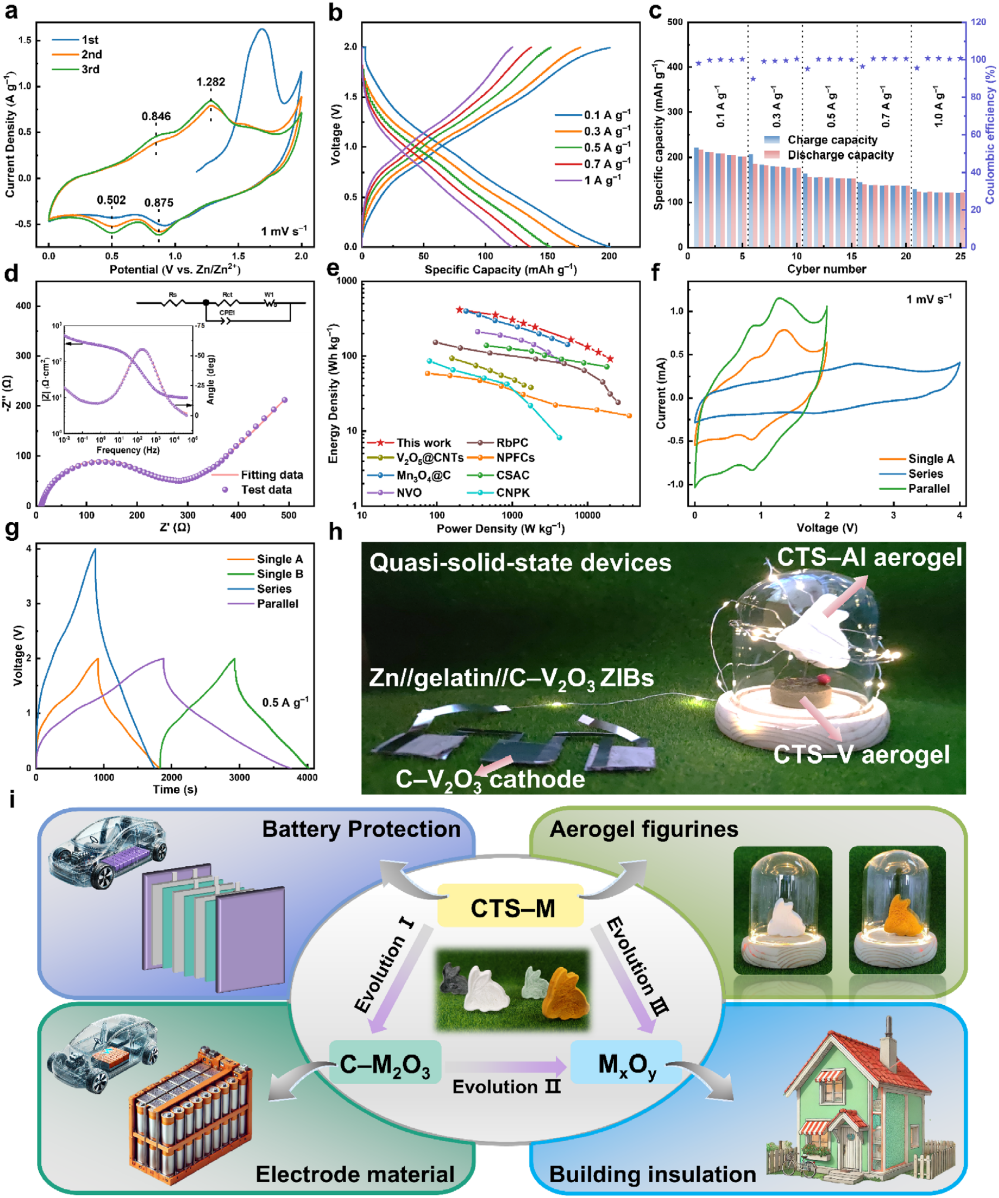
Electrochemical performance of quasi‐solid‐state Zn//gelatin//C–V_2_O_3_ ZIBs and application potentials of the CTS–M‐derived aerogels. (a) CV curves for the first three cycles at 1 mV s^−1^. (b) GCD profiles at different current densities. (c) Rate performance. (d) Nyquist plot with inset showing the equivalent circuit model and Bode plot. (e) Ragone plot comparison with previously reported electrode materials. CV (f) and GCD (g) curves of two quasi‐solid‐state ZIBs connected in series and parallel. (h) Photograph of 20 LED string lights embedded in CTS–M‐derived aerogel crafts, powered by three series‐connected ZIBs. (i) Potential application scenarios of the CTS−M‐derived aerogels.

To further evaluate practical adaptability, quasi‐solid‐state Zn//gelatin//C–V_2_O_3_ ZIBs were constructed (Figure 5). The initial CV curves (Figure 5a) show two pairs of redox peaks (1.282/0.875 and 0.846/0.502 V) with good overlap after the first cycle, indicating rapid activation and reversibility. Corresponding GCD profiles (Figure S66) confirm stable voltage plateaus. The device delivers capacities of 201 mAh g^−1^ at 0.1 A g^−1^ and 123 mAh g^−1^ at 1 A g^−1^ with nearly 100% Coulombic efficiency (Figure 5b, c). Impedance analysis (Figure 5d, Figure S67) indicates increased resistance compared to aqueous systems, attributed to the gelatin electrolyte, yet the spectra remain well described by a standard *R*_s_−*R*_ct_−Warburg model (Figure 5d inset), suggesting stable interfacial kinetics. Scan rate‐dependent CV (Figure S68) yields *b*‐values of 0.67−0.70, indicating mixed charge storage. As shown in Figure S68d, the capacitive contribution increases with scan rate, supporting the observed rate performance in Figure 5b, c.

At a current density of 5 A g^−1^, the device maintains nearly 100% coulombic efficiency over 10 000 cycles and retains 80% of its initial capacity, demonstrating excellent long‐term cycling stability (Figure S69). The Ragone plots (Figure 5e), normalized to the cathode active mass, show a high energy density of 415 Wh kg^−1^ at 200 W kg^−1^ and 92 Wh kg^−1^ at ∼20 000 W kg^−1^, outperforming representative solid‐state ZIBs based on carbon materials [57, 66, 67, 68], vanadium oxides [69], and C/TMO composites [62, 64]. Combined with a stable cycling performance, the C–V_2_O_3_‐based cathode exhibits a competitive overall performance among reported quasi‐solid‐state ZIB cathodes (Table S6). To evaluate scalability, two Zn//gelatin//C–V_2_O_3_ ZIBs were connected in series and parallel configurations. As shown in Figure 5f, g, the series connection extends the voltage window to 4.0 V, while the parallel configuration prolongs the discharge duration. As a proof‐of‐concept demonstration, three series‐connected Zn//gelatin//C–V_2_O_3_ ZIBs were used to power a 20 LED array integrated within CTS–M aerogel sculpture (Figure 5h and Movie S3), illustrating the practical feasibility of the system. Figure 5i summarizes the application potential enabled by this precursor‐unified platform. Notably, all components demonstrated here originate from the same CTS–M precursor system. The CTS–M aerogels can serve as humidity‐responsive, mechanically compliant external thermal buffer layers for battery modules. Upon carbonization under an inert atmosphere, they are transformed into electrochemically active C–M_2_O_3_ aerogels for energy storage. Alternatively, calcination in air can yield pure metal oxide aerogels suitable for high‐temperature thermal insulation. This stepwise divergence from a single molecular precursor highlights the versatility, internal consistency, and broad applicability of the CTS–M‐derived aerogel platform.

## 3 Conclusion

Here, we demonstrate that a single multiphase precursor can be directed through sequential structural evolution to yield materials with distinct yet complementary thermal regulation and electrochemical functionalities. Inspired by the natural principle of integrating chemically dissimilar phases into functional architectures, a universal one‐pot strategy is established to construct multifunctional aerogels that integrate adaptive thermal regulation with energy storage. The resulting CTS–M aerogels exhibit humidity‐triggered brittle‐to‐flexible transitions and reliable thermal insulation, enabling mechanical compliance and heat regulation across both high and subzero temperatures. These features make them promising as external thermal buffers or protective layers for battery modules, where they can simultaneously provide thermal insulation, mechanical cushioning, and adaptive heat buffering. Upon carbonization, the same precursor evolves into a second functional regime, where in situ growth of metal oxide nanocrystals within a conductive carbon framework generates C–M_2_O_3_ hybrid aerogels with coupled electrical conductivity and redox activity. As a representative system, C–V_2_O_3_‐based aqueous ZIBs deliver a high specific capacity (341 mAh g^−1^ at 0.1 A g^−1^), high energy density (656 Wh kg^−1^ at 200 W kg^−1^ and 178 Wh kg^−1^ at ∼20 000 W kg^−1^), and 85% capacity retention after 10 000 cycles, outperforming representative carbon–metal oxide composites. Collectively, this work demonstrates a design paradigm in which multifunctionality emerges from controlled material evolution rather than additive integration of disparate components. Moreover, an alternative oxidative pathway could further convert these hybrids into pure metal oxide aerogels, underscoring the compositional tunability of the precursor system. The demonstrated evolution from hygroscopic adaptability to electrochemical activity provides a scalable framework for the development of next‐generation self‐adaptive aerogels for energy and multifunctional device applications.

## Funding

This work was supported by the Natural Science Foundation of Shanghai (24ZR1470600).

## Conflicts of Interest

A patent for the metal chloride–chitosan aerogels and metal oxide–carbon composite aerogels (ZL 202511653722.1) has been filed on behalf of Tongji University. X. Z, X. W., and J. S. are inventors on the patent application.

## Supporting information




**Supporting File 1**: adma73935‐sup‐0001‐SuppMat.docx.

**Supporting File 2**: adma73935‐sup‐0002‐MovieS1.mp4.

**Supporting File 3**: adma73935‐sup‐0003‐MovieS2.mp4.

**Supporting File 4**: adma73935‐sup‐0004‐MovieS3.mp4.

## Data Availability

The data that support the findings of this study are available from the corresponding author upon reasonable request.
