# Supplementary material for: Evolution‐Driven Bio‐Composite Aerogels for Self‐Adaptive Thermal Regulation and Energy Storage
Source: Adv Mater. 2026 Jul 21;38(45):e73935. doi: 10.1002/adma.73935 (PMC13471765; doi:10.1002/adma.73935)
Supplement: Supplementary file 1 [file ADMA-38-e73935-s001.docx]

Supporting Information

**Evolution-Driven Bio-Composite Aerogels for Self-Adaptive Thermal Regulation and Energy Storage**

Xiaoxue Zhang^1^, Xiaodong Wang^1^*, Jianping Zeng^2^, Zhihua Zhang^1^, Shanyu Zhao^3^*, Jun Shen^1^

Xiaoxue Zhang, Xiaodong Wang, Zhihua Zhang, Jun Shen

School of Physics Science and Engineering, Tongji University, Shanghai, China
E-mail: xiaodong_wang@tongji.edu.cn (X. Wang)

Jianping Zeng

Integrated Systems Laboratory, Department of Information Technology and Electrical Engineering, ETH Zurich, Zurich, Switzerland

Shanyu Zhao
Laboratory for Building Energy Materials and Components, Swiss Federal Laboratories for Materials Science and Technology, Empa, Dübendorf, Switzerland

E-mail: shanyu.zhao@empa.ch (S. Zhao)

**Experimental Methods**

**Materials**

Aluminum chloride hexahydrate (AlCl_3_·6H_2_O), chromic chloride hexahydrate (CrCl_3_·6H_2_O), ferric chloride hexahydrate (FeCl_3_·6H_2_O), vanadium chloride (VCl_3_), chitosan (5–20 mPa·s, 20–100 mPa·s, 200–600 mPa·s), zinc sulfate heptahydrate (ZnSO_4_·7H_2_O), polyvinylidene difluoride (PVDF), carbon black (CB), N-Methyl-2-pyrrolidone (NMP) and deionized water were obtained from Sinopharm Chemical Reagent Co., Ltd. Gelatin was purchased from Shanghai Macklin Biochemical Technology Co., Ltd.

**Synthesis of CTS–M aerogels**

Initially, 0.6 g of chitosan was dissolved in 30 mL of 0.1 M MCl_3_ (M = Al, V, Cr, Fe) aqueous solution to form the CTS–MCl_3_ sol. The sol was frozen at −18 °C for 12 h and subsequently freeze-dried to yield the CTS−M aerogel. For comparison, 30 mL of 0.18 M acetic acid solution was prepared, in which 0.6 g of chitosan was dissolved. After undergoing the same freezing and freeze-drying process, pure CTS aerogel was obtained. For clarity, CTS–M–D and CTS–M–W are used throughout the text to denote the dry and moisture-conditioned states of CTS−M aerogels, respectively.

**Synthesis of C−M_2_O_3_ aerogels**

The C–M_2_O_3_ aerogel was obtained by calcining CTS–M aerogel under N_2_ atmosphere (300 mL min⁻^1^), following a two-step process of 90 °C for 3 h and Y °C (X=700, 800, 900, or 1000 °C) for 2 h, with a heating rate of 5 °C/min. The carbonized samples are denoted as C–M_2_O_3_–X–Y, where X (T, G, or B) refers to the precursor color (tan, green, or blue) and Y represents the carbonization temperature (e.g., C–V_2_O_3_–G–900 indicates the green-derived CTS–V sample carbonized at 900 °C).

**Characterization**

The mass and size (diameter and height) of the all aerogels were measured by an analytical balance (ES1205A, China) and a ruler, respectively, with density calculated according to formula (1). The surface morphology and elemental distribution of all samples were characterized using a SEM (Zeiss Gemini 300, Germany) coupled with an EDS (Oxford Xplore, UK). Prior to formal testing, the samples were sprayed with gold for 45 s using a sputter coater (Quorum SC7620, UK). The microstructure of the samples was analyzed by transmission electron microscopy (TEM, Thermo Fisher Talos F200X, USA). The crystal structure and phase analysis were determined via X-ray diffractometer (XRD, Bruker D2 Phaser, Germany) with a scanning rate of 10 °/min in the range of 5**–**80°. The chemical composition and elemental states of the sample surface were detected by X-ray photoelectron spectrometer (XPS, Thermo Scientific K-Alpha+, USA). The microstructure of CTS–M aerogels before and after moisture sorption was investigated by small angle X-ray scattering (SAXS, Xenocs Xeuss 2.0, France), where the corresponding X-ray wavelength of the copper target was 1.54 Å and the distance between the sample and the detector was fixed at 1185 mm. The composition and thermal stability of the aerogels were assessed by thermal gravimetric analyzer (TA TGA 550, USA) under an air atmosphere, with the temperature ramped from 20 °C to 800°C at a heating rate of 10°C/min. The nitrogen adsorption-desorption isotherms of C–V_2_O_3_ aerogels were measured using a fully automatic specific surface area and porosity analyzer (Micromeritics ASAP 2460, USA). The specific surface area and pore size distribution were calculated from the Brunauer-Emmett-Teller (BET) model and Barrett-Joyner-Halenda (BJH) method, respectively. Before testing, the samples were degassed at 120 °C for 12 h under vacuum. The composition and molecular structure of the samples were analyzed using a microscopes Raman spectrometer (LabRAM HR-800, France) with a laser wavelength of 514 nm. The functional groups and chemical bonds were identified via Fourier transform infrared spectrometer (FTIR, Bruker Tensor 27, Germany). The compressive cycling performance of cylindrical aerogels and the three-point bending performance of sheet-like aerogels were tested using an electronic universal testing machine (Shimadzu AGS-X, Japan), where the stress and strain for the three-point bending test were calculated according to formulas (2) and (3). The temperature changes on the back of the samples during heating or cooling was recorded by an infrared thermal imager (Fotric 626–L28, USA), with a microcomputer-controlled heating platform (JF966-2020, China) and a self-equipped liquid nitrogen device serving as the heat sources (110 °C) and cold sources (−196 °C), respectively. Due to the limited detection range of the infrared thermal imager, the cold plate temperature was only registered −26.7 °C. Measurements were conducted for 360 s after the temperature of the hot or cold plate reached a steady state. The thermal conductivity of the samples at room temperature was determined using a thermal constants analyzer (Hot Disk TPS 2500 S, Sweden) equipped with a Kapton-5501 sensor.

$\rho=\frac{m}{V}=\frac{m}{\left(\pi d^{2}h/4\right)}$ (1)

where$\rho$ represents density (g cm^–3^), *m* represents mass (g), *V* represents volume (cm^3^), *d* represents diameter (cm), and *h* represents height (cm).

$\sigma=\frac{3}{2}\cdot\frac{LF}{WT^{2}}$ (2)

$\varepsilon=\frac{6T}{L^{2}}\cdot\Delta l$ (3)

where$\sigma$ represents stress (Pa), *L* represents the distance between the lower support points (m), *F* represents load (N), *W* represents the length of the aerogel sheet (m), *T* represents the thickness of the aerogel sheet (m), $\varepsilon$represents strain (%) and$\Delta l$ represents bending deflection (m).

**Fog-harvesting measurements**

The moisture sorption tests were carried out in an indoor environment with open windows and fluctuating temperature and humidity to mimic practical vehicle conditions. 0.5 g of dry CTS–Al aerogel, CTS–Cr aerogel, CTS–Fe aerogel, CTS powder, AlCl_3_·6H_2_O powder, CrCl_3_·6H_2_O powder, and FeCl_3_·6H_2_O powder were placed in open petri dishes to absorb the moisture from the air. The samples were weighed periodically, and their moisture sorption were calculated according to formula (4). The above processes were repeated until the mass of each sample stabilized, signifying the cessation of moisture sorption.

$Moisture\text{ }absorption=\left(m_{n}-m_{0}\right)/m_{0}$ (4)

where *m*_0_ represents the mass of the dry samples, and *𝑚*_𝑛_ represents the mass of these samples after moisture sorption.

**Compression**–**release tests after moisture sorption–evaporation cycling**

The compression–release test after moisture sorption–evaporation cycling was conducted as follows. Dry CTS–M aerogels were placed in an open Petri dish and exposed to ambient air until reaching an atmospheric moisture-sorption equilibrium, as indicated by the nearly unchanged sample mass upon further exposure. Subsequently, compression–release tests at 30% strain were carried out using an electronic universal testing machine. Immediately after testing, the samples were dried in a 60 °C oven for 2 h to remove the absorbed moisture. The above moisture sorption and drying operations were repeated. Compression–release tests were performed after every 10 cycles, and the entire process was repeated for 100 cycles.

**Moisture-sorption tests for determining the brittle-to-flexible transition threshold**

To determine the critical moisture-sorption condition for the brittle-to-flexible transition of CTS–M aerogels, two complementary experiments were conducted. First, time-dependent moisture-sorption experiments were carried out under a daily indoor environment with open windows and fluctuating temperature and humidity. Dry CTS–M aerogels were exposed to air for 0, 1, 3, and 7 h, respectively. At each time point, the samples were weighed, and the moisture uptake was calculated according to Eq. (4). Immediately after weighing, the samples were subjected to uniaxial compression tests. By correlating the corresponding stress–strain behavior with the measured moisture uptake at different exposure times, the evolution from brittle to flexible deformation was determined. Second, controlled-humidity experiments were performed using saturated salt solutions to construct different relative-humidity environments. Saturated LiCl, Mg(NO_3_)_2_, NaCl, and KNO_3_ solutions were separately placed in sealed containers to establish different humidity conditions. Dry CTS–M aerogels were then placed in these environments for 24 h. After 24 h of moisture sorption, the samples were weighed, and the moisture uptake was calculated according to formula (4). The samples were then subjected to uniaxial compression tests. By comparing the mechanical responses of samples exposed to different humidity conditions, the critical moisture-sorption range corresponding to the brittle-to-flexible transition was further identified. Taken together, although the two experiments used moisture-sorption time and relative humidity as different external variables, both fundamentally regulated the absorbed moisture content of the aerogels. Therefore, these two sets of experiments jointly established the critical moisture-uptake threshold governing the brittle-to-flexible transition of CTS–M aerogels.

**Temperature-dependent compression modulus measurements**

The temperature-dependent compression modulus measurements were carried out as follows. Dry CTS–M aerogels were first placed in a daily indoor environment with open windows and fluctuating temperature and humidity for 24 h to absorb moisture from the air. After 24 h, the samples were weighed, and their moisture uptake was calculated according to formula (4). The obtained moisture-conditioned samples were denoted as CTS–M–W. Subsequently, the CTS–M–W aerogels were separately placed on constant-temperature heating stages at 20, 40, 60, and 80 °C for 5 min to simulate the practical contact condition with heat-generating devices. The holding time of 5 min was selected according to the thermal equilibration time determined from the heat-transfer tests on the hot plate (110 °C) and cold plate (−196 °C). Immediately after thermal conditioning, uniaxial compression tests were performed at the corresponding temperature. The compressive modulus values were then calculated from the corresponding stress–strain curves. In addition, the moisture uptake of the three CTS–M aerogels after 24 h exposure was recorded before heating, so that the influence of the initial absorbed moisture content on the brittle-to-flexible transition under different temperature conditions could also be analyzed.

**Moisture sorption–evaporation cycling tests**

The moisture sorption–evaporation cycling tests were carried out under both dynamic ambient conditions and a controlled-humidity environment. For the dynamic ambient cycling test, dry CTS–M aerogel samples were placed in an indoor environment with open windows and fluctuating temperature and humidity to absorb moisture from the air. The sample mass was recorded every 4 h, and the moisture uptake was calculated according to formula (4). A period of 24 h was defined as one moisture-sorption cycle. After each cycle, the samples were dried in a 60 °C oven to remove the absorbed moisture and then reused for the next cycle. The above procedure was repeated for 10 cycles. For the controlled-humidity cycling test, dry CTS–M aerogel samples were placed in a sealed humidity-controlled environment constructed using saturated salt solution. The sample mass was recorded every 4 h, and the moisture uptake was calculated according to formula (4). A period of 24 h was defined as one moisture-sorption cycle. After each cycle, the samples were dried in a 60 °C oven to remove the absorbed moisture and then reused for the next cycle. The above procedure was repeated for 5 cycles. By comparing the moisture uptake behavior over repeated cycles under these two conditions, the cycling stability of the moisture sorption capability of CTS–M aerogels was evaluated.

**Leakage verification after moisture sorption**

The leakage verification test was carried out as follows. Dry CTS–M aerogels were first placed in an indoor environment with open windows and fluctuating temperature and humidity to absorb moisture from the air. After the sample mass became stable, the obtained moisture-conditioned samples were denoted as CTS–M–W. Subsequently, the CTS–M–W aerogels were placed on dry weighing paper and manually compressed. After compression, the samples were removed, and the paper surface was visually examined for any wet marks or liquid traces. The same procedure was performed for CTS–Al–W, CTS–Cr–W, and CTS–Fe–W samples. This test was used to determine whether the absorbed moisture existed as free liquid that could be released under external deformation.

**Electrochemical measurements**

The C–V_2_O_3_ cathode was prepared by coating the graphite paper with a slurry composed of C–V_2_O_3_, CB and PVDF in a mass ratio of 8:1:1, followed by drying. The gelatin electrolyte was synthesized by dissolving 2 g of gelatin in 10 mL of deionized water in a 60°C water bath, then adding 10 mL of 2M ZnSO_4_ to it and stirring it for 1h. The gelatin hydrogel electrolyte was obtained after cooling at room temperature. Using C–V_2_O_3_ as the cathode and zinc foil (0.15–0.25 mm) as the anode, aqueous ZIBs were assembled when the electrolyte was 2M ZnSO_4_, and all-solid-state ZIBs were obtained when the electrolyte was the gelatin hydrogel. The electrochemical properties of ZIBs, including cyclic voltammetry (CV), galvanostatic charge-discharge (GCD), electrochemical impedance spectroscopy (EIS), and long-cycle performance were evaluated by the electrochemical workstation (CHI660A, China) and the LAND testing system (LAND CT2001A, China).

$Q=\frac{I}{m}\left(\frac{\Delta t}{3.6}\right)$ (5)

$CE=\frac{Discharge Capacity }{Charge\text{ }Capacity}*100\%$ (6)

where *Q* represents the specific capacity (mAh g^−1^), I represents the discharge current (A), m represents the mass of the active material (g), and$\Delta t$ represents the discharge time (s).

**Figure S1.** FTIR spectra of CTS–M (M = Al^3+^, Cr^3+^, Fe^3+^, V^3+^) aerogels and pristine CTS. (a) Full-range spectra; (b) Enlarged view of the secondary hydroxyl peak at the C3 position.

Figure S1a compares the FTIR spectra of pristine CTS and CTS–M aerogels to elucidate the potential coordination mechanism between trivalent metal ions and chitosan. All samples exhibit a characteristic C−H stretching vibration peak near 2918 cm^−1^, indicating that the introduction of metal ions does not disrupt the chitosan backbone. The broad absorption band at 3441 cm^−1^, attributed to the O−H and N−H stretching vibrations of CTS, undergoes a noticeable red shift to 3416–3431 cm^−1^ in the CTS–M samples. This shift suggests that the metal ions coordinate with the surface hydroxyl and amino groups of chitosan, thereby weakening the original hydrogen bonding network and reducing the vibrational frequency of O−H and N−H groups. Additionally, the N−H bending vibration peak at 1641 cm^−1^ shifts to lower wavenumbers (1622–1630 cm^−1^) in the CTS–M aerogels, further confirming the coordination interaction between the lone pair electrons of the nitrogen atom and the metal ions. Moreover, the characteristic peak of the secondary hydroxyl group at the C3 position (~1074 cm^−1^) shows reduced intensity and a slight blue shift in the CTS–M samples (Figure S1b), suggesting its involvement in metal coordination. These spectral changes collectively demonstrate that both −OH and −NH_2_ groups in CTS engage in effective synergistic coordination with trivalent metal ions, validating the bioinspired design strategy proposed in this work.

**Figure S2.** XRD patterns of CTS–Al (a), CTS–Cr (b), CTS–Fe (c), and CTS–V (d) aerogels and their corresponding precursors (CTS and MCl_3_).

Figure S2 presents the XRD patterns of pristine chitosan (CTS), hydrated metal chloride precursors (MCl_3_·6H_2_O, M = Al, Cr, Fe, V), and the resulting CTS–M aerogels, aimed at further validating the coordination interactions between chitosan and trivalent metal ions (M^3+^). All metal chloride hydrates exhibit multiple sharp and intense diffraction peaks, consistent with their well-defined crystalline hydrate structures. Pristine CTS shows distinct diffraction peaks at 10.9° and 20.1°, attributed to crystalline ordering induced by intramolecular and intermolecular hydrogen bonding between amino and hydroxyl groups along the polymer chains. In contrast, all CTS–M aerogels display a broad amorphous halo, with no residual diffraction peaks corresponding to the CTS or MCl_3_·6H_2_O precursors. This indicates that the hydrogen-bonding interactions within the chitosan backbone are substantially disrupted during complexation, and that the metal ions no longer exist in a crystalline state but are instead homogeneously incorporated into the polymer matrix via coordination. Together with the FTIR results, these findings confirm that both −NH_2_ and −OH groups of chitosan participate in coordination with M^3+^ ions, leading to the formation of uniform amorphous networks. This result strongly supports the rationality of the bioinspired coordination strategy proposed in this study and is highly consistent with the coordination-induced spectral shifts observed in FTIR analysis and the amorphous structural features revealed by XRD.

**Figure S3.** Photos of bioinspired rabbit-shaped CTS–M and C–M_2_O_3_ aerogels. Inset: photo of CTS–Fe aerogel resting on a flower.

**Figure S4.** SEM images of CTS–Cr aerogel before (a–d) and after (e–h) moisture sorption (D = dry, W = moisture-conditioned).

After exposure to ambient humidity, the CTS–Cr aerogels exhibit significant morphological changes compared to their dry-state counterparts. SEM images show a marked reduction in visible open pores, obvious swelling of the fibrous skeleton, and densification of the overall structure (Figure S4e–h). Additionally, localized regions outlined in red in Figure S4e–g display the emergence of lamellar surface wrinkles after moisture sorption, indicating structural softening and laying the foundation for the aerogel’s environmentally adaptive mechanical response.

**Figure S5.** SEM image (a) and EDS elemental mapping (b) of CTS–Cr aerogel.

**Figure S6.** XRD patterns of C–Al_2_O_3_, C–Cr_2_O_3_, and C–Fe_2_O_3_ aerogels.

For all three samples, a broad diffraction feature centered at ~20° is observed, which can be assigned to the (002) plane of amorphous carbon ^[1]^, confirming the successful carbonization of the chitosan framework. Meanwhile, distinct crystalline phases corresponding to the respective metal oxides are clearly identified after pyrolysis. Specifically, the diffraction peaks of C–Al_2_O_3_ aerogel can be indexed to γ-Al_2_O_3_ corresponding to the (220), (311), (400), and (440) planes (PDF#10-0425) ^[2]^. C–Cr_2_O_3_ aerogel exhibits characteristic peaks of Cr_2_O_3_, including the (012), (104), (110), and (113) planes (PDF#38-1479) ^[3]^. For C–Fe_2_O_3_ aerogel, the observed peaks are consistent with Fe_2_O_3_, mainly assigned to the (220), (311), (511), and (440) planes (PDF#39-1346) ^[4]^. Importantly, these results are consistent with the structural evolution observed in the C–V_2_O_3_ system, confirming that the coordination-derived CTS–M precursors can be generally transformed into carbon/metal-oxide hybrid aerogels upon pyrolysis, regardless of the specific metal species. This additional evidence further supports the generality and transferability of the proposed two-stage evolution strategy.

**Figure S7.** SEM images of C–Al_2_O_3_ (a), C–V_2_O_3_ (b), C–Cr_2_O_3_ (c), and C–Fe_2_O_3_ (d) aerogels after carbonization at 800 °C. Please note that the C–V₂O₃ sample shown in Figure S7b was selected for the subsequent investigations and performance evaluations presented in Figures S52d–f, S56e–h, and S59e–h.

From the SEM comparison (Figure S7), it is evident that the identity of the metal ions plays a key role in governing the morphology evolution during carbonization. For the Al, Cr, and Fe systems, the original fibrous framework structure of CTS is largely preserved after pyrolysis, forming conductive carbon skeletons with metal oxide nanoparticles anchored on the surface. As a result, C–Al_2_O_3_, C–Cr_2_O_3_, and C–Fe_2_O_3_ aerogels predominantly exhibit fibrous carbon frameworks decorated with metal oxide particles. In contrast, the V-containing system shows a distinctly different evolution pathway. As discussed in the main text, the CTS–V precursor initially exhibits a similar fibrous framework decorated with surface particles, but subsequently undergoes further structural reorganization into spherical aggregates. After pyrolysis, this evolution leads to a hierarchical granular/spherical C–V_2_O_3_ architecture with surface-decorated V_2_O_3_ nanocrystals, clearly different from the framework-preserving morphologies observed in the Al, Cr, and Fe systems. These results indicate that different trivalent metal ions not only influence the initial coordination environment, but also determine the subsequent morphology-evolution mode during pyrolysis.

**Figure S8.** (a) CV curves of C–M_2_O_3_ aerogels at 10 mV s^−1^, (b) GCD curves of C–M_2_O_3_ aerogels at 0.5 A g^−1^.

The electrochemical comparison (Figure S8) further reveals a clear dependence on the metal species. At a scan rate of 10 mV s^−1^, C–V_2_O_3_ aerogel exhibits the largest CV area and the most pronounced redox features. Consistently, at a current density of 0.5 A g^−1^, C–V_2_O_3_ aerogel delivers the longest discharge time and high specific capacity of 226 mAh g^−1^, significantly outperforming the other three samples. In comparison, C–Cr_2_O_3_ and C–Fe_2_O_3_ aerogels show moderate performance, while C–Al_2_O_3_ aerogel exhibits relatively weak electrochemical activity. We attribute this trend to the combined effects of composition and structure. Structurally, the reconstructed granular/spherical architecture of C–V_2_O_3_ aerogel provides more accessible interfaces, better dispersion of active sites, and shorter ion/electron transport pathways. From a compositional perspective, vanadium oxides are widely recognized as favorable cathode materials for aqueous zinc-ion batteries due to their multivalent redox chemistry, high charge-storage capability, and fast Zn^2+^ storage kinetics ^[5]^, whereas Al_2_O_3_ is relatively electrochemically inert and Cr_2_O_3_ and Fe_2_O_3_ exhibit comparatively limited activity under similar conditions ^[6]^.

**Figure S9.** SEM images and corresponding elemental mapping of CTS–V aerogels with different colors. (a–d) CTS–V–T, (e–h) CTS–V–G, (i–l) CTS–V–B.

SEM images (Figure S9a–c, e–g, i–k) revealed that the as-prepared aerogels exhibited continuous and smooth surface morphologies prior to carbonization. Elemental mapping (Figure S9d, h, l) further confirmed that C and O were concentrated within the fibrous or spherical frameworks, while V was uniformly distributed throughout the matrix, without observable agglomeration or depletion zones. This indicates that the aerogel skeleton was composed primarily of the C/O-rich CTS backbone, with V^3+^ uniformly embedded via coordination—providing the basis for subsequent in situ growth and dispersion of metal oxides.

HRTEM images (Figure S10) revealed clear lattice fringes corresponding to the (104), (110), (012), and (113) planes of V_2_O_3_, confirming the formation of highly crystalline V_2_O_3_ nanoparticles on the carbon skeleton. Between these nanocrystals, amorphous regions lacking lattice fringes were also evident, consistent with the formation of disordered carbon from carbonized CTS, further supporting the tight integration between the carbon network and V_2_O_3_ phase.

**Figure S10.** TEM and STEM-EDS analysis of C–V_2_O_3_ aerogels. (a, b) HRTEM images, (c) TEM image, (d) HAADF-STEM image, (e–h) STEM-EDS mapping of V, O, and C.

**Figure S11.** Local XRD magnification of CTS–V aerogels before and after carbonization. (a) CTS–V aerogels, (b) C–V_2_O_3_ aerogels.

As shown in Figure S11a, none of the CTS–V samples exhibited crystalline diffraction peaks prior to carbonization; instead, a broad and weak halo was observed in the 2θ range of 15°–23° (highlighted by the cyan dashed box), indicative of typical amorphous polymeric aerogels. The enlarged XRD patterns in Figure S11b reveal a broad and weak feature in the 2θ range of 16°–24° in all carbonized samples, which can be attributed to the (002) diffraction of amorphous carbon. The presence of this peak suggests that although V_2_O_3_ crystallization occurred, the original chitosan backbone partially converted into structurally disordered, conductive carbon domains.

**Figure S12.** Thermogravimetric (TG) and derivative thermogravimetric (DTG) curves of C–V_2_O_3_ aerogels prepared at different carbonization temperatures. (a) 700 °C, (b) 800 °C, (c) 900 °C, and (d) 1000 °C.

Figure S12 provides detailed TG–DTG profiles of C–V_2_O_3_ aerogels prepared at different carbonization temperatures. Overall, the 700 and 800 °C samples exhibit a three-stage mass-evolution behavior (Figure S12a, b), whereas the 900 and 1000 °C samples show a more simplified two-stage weight-loss process (Figure S12c, d). The initial low-temperature weight loss below 200 °C is mainly associated with the removal of physically adsorbed water and weakly bound species. For the 700 °C sample, the intermediate weight-loss region of 200–350 °C can be assigned to the decomposition/oxidation of residual organic species and thermally labile carbonaceous components, together with the early evolution of vanadium oxide species. The major weight-loss stage at 350–500 °C primarily corresponds to the combustion of the carbon framework in air, accompanied by the further oxidation of vanadium oxide species toward higher-valence oxides. The 800 °C sample follows a similar three-stage pattern, but displays a more distinct early-stage mass-loss behavior and a shifted DTG response. As further revealed by temperature-resolved XRD and Raman analyses (Figure S13), this anomaly originates from a differentiated early oxidation pathway involving a more pronounced transient V_3_O_7_ intermediate and a relatively more defective residual carbon framework, rather than the formation of a unique final oxidation product. Upon further heating, all samples eventually evolve toward V_2_O_5_. Therefore, the slight mass increase around 500–550 °C can be reasonably attributed to oxygen uptake during the further oxidation of residual low-valence vanadium oxide species toward V_2_O_5_ in air ^[7-10]^.

**Figure S13.** Temperature-resolved structural evolution of C–V_2_O_3_ aerogels during calcination in air. (a) XRD patterns of C–V_2_O_3_–700, C–V_2_O_3_–800, C–V_2_O_3_–900, and C–V_2_O_3_–1000 after heating to 350 °C. (b) Enlarged XRD pattern of C–V_2_O_3_–700 in the selected 2θ range at 350 °C. (c) Raman spectra of the corresponding samples after heating to 350 °C. (d–f) XRD patterns after heating to 400 °C (d), 450 °C (e), and 500 °C (f).

**Figure S14.** Mass ratio of carbon to V_2_O_3_ (C/V_2_O_3_) in C–V_2_O_3_ aerogels prepared at different carbonization temperatures.

For quantitative comparison, the C/V_2_O_3_ mass ratio was estimated from the TG results (Figure S14). Since the final residue after TG analysis in air mainly corresponds to fully oxidized V_2_O_5_ rather than the original V_2_O_3_ phase, the V_2_O_3_ content was back-calculated using the molar-mass ratio between V_2_O_3_ and V_2_O_5_. Meanwhile, the low-temperature moisture loss was excluded from the carbon-content estimation. Based on this analysis, the C/V_2_O_3_ mass ratios of the samples carbonized at 700, 800, 900, and 1000 °C are estimated to be 0.90, 0.73, 0.53, and 0.29, respectively. This continuous decrease indicates that increasing carbonization temperature gradually reduces the relative carbon fraction while increasing the proportion of V_2_O_3_ in the hybrid aerogels. Correlating this compositional evolution with the electrochemical performance, the 900 °C sample exhibits an intermediate C/V_2_O_3_ mass ratio of 0.53, providing a favorable balance between conductive carbon and redox-active V_2_O_3_. This optimized balance contributes to efficient electron transport, active Zn^2+^-storage-site utilization, and structural stability, thereby leading to the best electrochemical performance among the investigated samples. Therefore, these results not only confirm the tunability of carbon content and thermal stability via carbonization temperature, but also provide strategic insights for tailoring composite aerogel architectures toward enhanced electrochemical properties.

**Figure S15.** Point-resolved Raman analysis of the C–V_2_O_3_–G aerogel. (a) Raman optical micrograph with marked test points (Sp1–Sp5). (b) Raman spectra collected at the corresponding positions.

Figure S15 further presents the site-specific Raman analysis of the C–V_2_O_3_–G aerogel acquired from different microscopic regions. As shown in Figure S15a, the spectra collected from Sp1 to Sp3, located in the dark spherical core region, exhibit prominent D and G bands, confirming that these regions correspond to the carbon framework. In contrast, the spectra from Sp4 and Sp5, located in the outer purple-coated regions, show no discernible D or G bands but display a characteristic V_2_O_3_ E_g_ vibrational mode at approximately 570 cm^−1^, confirming the presence of crystalline V_2_O_3_ nanoparticles. These observations are consistent with the SEM/TEM results, further supporting the proposed structural model in which V_2_O_3_ nanoparticles are uniformly distributed on the surface of the spherical carbon framework.

**Figure S16.** XPS survey spectra of CTS–V aerogels with different colors before (a) and after (b) carbonization.

The survey spectra (Figure S16a) reveal that, all CTS–V samples display distinct C 1s, O 1s, N 1s, V 2s, V 2p, Cl 2s and Cl 2p peaks. The C, O and N signals originate from the chitosan backbone, while the Cl and V signals derive from the VCl_3_ precursor, confirming effective coordination between the polymer framework and VCl_3_. After carbonization (Figure S16b), the Cl peaks vanish completely, indicating full volatilization of chlorine under the high-temperature inert atmosphere and a corresponding increase in the chemical purity of the C–V_2_O_3_ aerogel. In contrast, the N 1s signal remains detectable, evidencing partial retention and doping of nitrogen within the carbon matrix (Figure S16b). The V signal intensifies markedly upon carbonization owing to conversion of coordinated V^3+^ into surface-enriched crystalline V_2_O_3_, which enhances its XPS detectability. Concurrently, the integrated intensity ratio of C 1s to O 1s increases from 0.67 to 1.16 (Figure S16), reflecting cleavage of organic functional groups and formation of the carbon framework during heat treatment.

**Figure S17.** High-resolution XPS spectra comparison of CTS–V–G and C–V_2_O_3_–G aerogels. (a, e) C 1s, (b, f) O 1s, (c, g) V 2p, and (d, h) N 1s.

**Figure S18.** High-resolution XPS spectra comparison of CTS–V–T and C–V_2_O_3_–T aerogels. (a, e) C 1s, (b, f) O 1s, (c, g) V 2p, and (d, h) N 1s.

**Figure S19.** High-resolution XPS spectra comparison of CTS–V–B and C–V_2_O_3_–B aerogels. (a, e) C 1s, (b, f) O 1s, (c, g) V 2p, and (d, h) N 1s.

High-resolution C 1s spectra (Figure S17–S19a, e) for the precursors can be deconvoluted into C−C (~ 284.8 eV), C−O (~ 286.5 eV) and C=O (~ 288.5 eV) contributions, corresponding to the chitosan backbone, hydroxyls and residual amide groups, respectively. After carbonization, the C−C component remains dominant, whereas C−O and C=O intensities diminish substantially, corroborating preservation of the carbon skeleton and partial removal of oxygenated groups. The O 1s spectra (Figure S17–S19b, f) of the three color-variant CTS–V samples prior to carbonization can be fitted with three components at 533.3–533.7 eV, 532.1–532.7 eV and 530.6–531.5 eV, assigned to C=O, C−O and V^3+^−O coordination bonds with chitosan hydroxyls, respectively ^[11]^. After carbonization, pronounced peak shifts and intensity redistribution occur. A strong peak at ~ 530.4–530.5 eV is attributable to V−O bonds in crystalline V_2_O_3_, confirming in situ transformation of the vanadium complex into an oxide phase. Notably, an intense C−O−V feature emerges at ~ 531.9–532.0 eV, implying formation of bridging bonds between oxygen in the carbon matrix and surface vanadium oxides, further evidencing in situ growth of V_2_O_3_ and robust interfacial coupling with the carbon scaffold. The V 2p spectra (Figures S17–S19c, g) trace the valence evolution of vanadium. In the precursors, the characteristic V^3+^ doublet appears at 516.2–516.7 eV (V 2p_3/2_) and 523.7–524.1 eV (V 2p_1/2_), indicating that V is coordinated in a stable +3 state. Minor binding-energy variations among the color-variant samples likely reflect changes in the local coordination environment of V^3+^. Post-carbonization, two main peaks at 517.3–517.4 eV (V 2p_3/2_) and 525.0 eV (V 2p_1/2_) match well with crystalline V_2_O_3_, confirming effective conversion of the V complexes into V_2_O_3_. A small shoulder at ~515.8–516.2 eV is assigned to V^2+^, arising from partial reduction of V^3+^ in the high-temperature carbonaceous environment ^[12-15]^. The resulting V^3+^/V^2+^ mixture implies generation of oxygen vacancies, which can enhance electronic transport and interfacial reactivity. The N 1s spectra (Figures S17–S19d, h) of the precursors can be deconvoluted into two characteristic peaks. The peak at ~399.2–400.1 eV is assigned to –NH_2_ groups from the chitosan backbone, whereas the higher-binding-energy peak at ~401.8–402.0 eV corresponds to –NH_3_^+^/coordinated N species ^[16]^. These features indicate partial protonation of the amino groups together with coordination between V^3+^ ions and nitrogen-containing groups during precursor formation. After carbonization, the N 1s spectra evolve into two characteristic peaks located at ~398.4–398.6 eV and ~401.4 eV, corresponding to pyridinic-N and graphitic-N, respectively ^[17, 18]^. The persistence of these nitrogen species confirms successful N doping of the carbon framework during pyrolysis. In particular, pyridinic-N provides electrochemically active sites by regulating the local charge distribution, whereas graphitic-N enhances the electrical conductivity of the carbon matrix by facilitating electron transport ^[17, 19]^.

**Figure S20.** Valence band XPS spectra of CTS–V aerogels with different color states before and after carbonization. (a) Full-range spectra of all samples, (b) enlarged spectra of CTS–V and C–V_2_O_3_.

Valence-band spectra (Figure S20) further reveal the global electronic-structure evolution. Prior to carbonization, three broad features reflect the disordered overlap of hybrid orbitals from C, O and V in the amorphous organic framework. After carbonization, the spectra simplify into sharper peak with significantly higher state density near the valence-band edge (Figure S20b), indicating transformation to an ordered graphitic carbon/V_2_O_3_ hybrid, conducive to enhanced conductivity and accelerated electron transfer.

**Figure S21.** Moisture sorption of MCl_3_ (M = Al, Cr, Fe), CTS aerogel, and CTS–M aerogels under dynamic ambient conditions, expressed as moisture uptake per unit dry mass (g g^−1^).

The standard Gibbs free energies of hydration at 298 K are –4525, –4190, and –4050 kJ·mol^−1^ for Al^3+^, Cr^3+^, and Fe^3+^, respectively [14]. The much stronger hydration of Al^3+^ is consistent with the highest moisture uptake observed for CTS–Al aerogel.

**Figure S22.** Macroscopic morphology of samples before and after moisture sorption. (a) MCl_3_ after moisture sorption, (b) CTS before and after moisture sorption, (c) CTS–M aerogel before moisture sorption, and (d) CTS–M aerogel after moisture sorption.

MCl_3_ undergoes severe deliquescence upon moisture exposure (Figure S22a), whereas CTS remains morphologically unchanged (Figure S22b), confirming that water sorption in CTS–M aerogels is primarily driven by the metal ions rather than the chitosan matrix. In contrast, the CTS–Cr aerogel retains its structural integrity after moisture sorption (Figure S22c, d), indicating not only enhanced water retention capability but also overcoming the instability of MCl_3_.

**Figure S23.** FTIR spectra of CTS–M aerogels before and after moisture sorption. (a–c) O−H stretching region, (d–f) full-range FTIR spectra.

FTIR spectra (Figure S23d–f) show that the hydrated CTS–M aerogels retain the major characteristic peaks of CTS, indicating that the chemical integrity of the polymer backbone is preserved after moisture sorption. To rigorously evaluate the evolution of hydrogen bonding, the O−H/N−H stretching vibration (~3400 cm^−1^) was normalized against the C−H stretching peak at 2918 cm⁻¹. The intensity ratio of C−H to O−H/N−H significantly decreased after moisture sorption: from 0.18 to 0.10 for CTS–Al, from 0.16 to 0.07 for CTS–Cr, and from 0.16 to 0.04 for CTS–Fe (Figure S23a–c). This downward trend indicates a marked increase in hydrogen-bond-related functionalities, confirming the efficient incorporation of water molecules and the reconstruction of the hydrogen-bonding network. These newly formed hydrogen bonds likely replace part of the rigid interchain hydrogen bonds, reduce intermolecular interactions, and enhance polymer chain mobility, thereby enabling a reversible transition from brittle to flexible states and establishing the structural basis for moisture-responsive mechanical softening.

**Figure S24.** Schematic illustration of the home-built setup for moisture sorption experiments under (a) constant humidity and (b) dynamic ambient humidity conditions.

**Figure S25.** Uniaxial compression stress–strain curves of CTS–Al (a, b) and CTS–Fe (c, d) aerogels after different time of moisture sorption under dynamic ambient condition.

**Figure S26.** Moisture uptake of CTS–Al and CTS–Fe aerogels under ambient condition.

Under ambient conditions, CTS–Al and CTS–Fe aerogels were exposed to air at room temperature for different durations (0, 1, 3, and 7 h), followed by uniaxial compression tests with simultaneous monitoring of moisture uptake. The results demonstrate that the brittle-to-flexible transition is not an instantaneous but evolves progressively with increasing moisture uptake. In the dry state (0 h), both aerogels exhibit typical brittle fracture behavior, characterized by abrupt stress drops in the stress–strain curves (Figure S25a, c). After 1 h exposure, limited moisture uptake occurs (0.17 g g^−1^ for CTS–Al aerogel and 0.09 g g^−1^ for CTS–Fe aerogel; Figure S26), leading to partial softening. However, the stress-strain curves still show discontinuous and multiple stress drops (Figure S25b, d), indicating that the structure remains in a transitional state with localized fracture event. With further moisture sorption, the mechanical response becomes significantly smoother and more continuous. At 3–7 h, the moisture uptake increases to 0.35–0.38 g g^−1^ (CTS–Al aerogel) and 0.23–0.24 g g^−1^ (CTS–Fe aerogel), as shown in Figure S26. Correspondingly, the stress–strain curves no longer exhibit fracture-dominated features, indicating the establishment of a flexible state. These results confirm that a sufficient level of absorbed moisture is required to fully suppress brittle fracture and enable flexible deformation. Based on the dynamic-ambient experiment, the brittle-to-flexible transition exhibits a threshold-like dependence on moisture uptake rather than occurring instantaneously. For CTS–Al aerogel, brittle fracture persists at a moisture uptake of 0.17 g g^−1^, whereas a predominantly flexible response emerges at moisture uptake ≥ 0.35 g g^−1^. A comparable evolution is observed for CTS–Fe aerogel (0.09 to 0.23 g g^−1^), confirming that a critical level of absorbed moisture is required to fully activate the flexible deformation regime.

**Figure S27.** Uniaxial compression stress–strain curves of CTS–Al (a), CTS–Cr (b), and CTS–Fe (c) aerogels after exposing at different relative humidity for 24 h. The curves of RH=0% refer to those of the dry samples.

**Figure S28.** Moisture uptake of CTS–Al (a), CTS–Cr (b), and CTS–Fe (c) aerogels after exposing at different relative humidity for 24 h.

To further determine whether brittle-to-flexible transition occurs above a specific humidity level, we conducted controlled-humidity experiments using saturated salt solutions, where RH of 17%, 48%, 70%, and 81% were simulated, respectively. In the dry state (0% RH), all three samples exhibit multiple abrupt stress drops during compression, indicating typical brittle fracture-dominated behavior (Figure S27). At low humidity (17% RH), only limited moisture uptake is observed (0.07 g g^−1^ for CTS–Al; 0.06 g g^−1^ for CTS–Cr, and 0.06 g g^−1^ for CTS–Fe), and all samples still exhibit relatively rigid mechanical responses with minor stress fluctuations, indicating incomplete transition (Figures S27 and S28). In contrast, at 48% RH, the moisture uptake increases significantly (0.26 g g^−1^ for CTS–Al, 0.22 g g^−1^ for CTS–Cr, and 0.21 g g^−1^ for CTS–Fe), and the stress–strain curves become smooth and continuous, indicating a transition to a flexible state. Further increases in humidity (70%–81% RH) lead to higher but nearly saturated moisture uptake (CTS–Al: 0.51 g g^−1^ at 70% RH, 0.57 g g^−1^ at 81 RH%; CTS–Cr: 0.47 g g^−1^ at 70% RH, 0.49g g^−1^ at 81% RH; CTS–Fe: 0.42 g g^−1^ at 70% RH, 0.45 g g^−1^ at 81% RH; Figure S28), with consistently stable flexible behavior.

Overall, a clear correlation was established among moisture sorption conditions (relative humidity and exposure time), moisture uptake, and mechanical state. Although relative humidity and exposure time are different external variables, both regulate the brittle-to-flexible transition through their effect on moisture uptake. Specifically, the flexible state emerges once the absorbed moisture reaches a critical range, which can be achieved either under sufficiently high relative humidity or after prolonged ambient exposure.

**Figure S29.** Cycling moisture sorption results of CTS–Al, and CTS–Cr aerogels under dynamic ambient condition (a, b) and constant humidity (RH≈68%) condition (c, d).

Two complementary cycling protocols were designed to evaluate the robustness of the aerogels. First, cycling tests were conducted under dynamic ambient condition. CTS–Al and CTS–Cr aerogels were directly exposed to air, and their mass was recorded every 4 h over a 24 h period to monitor moisture uptake. After each cycle, samples were dried at 60 °C and reused for subsequent cycles, for a total of 10 cycles. As shown in Figure S29a, b, the moisture uptake exhibits adaptive fluctuations both within and between cycles due to natural variations in environmental humidity. Despite these fluctuations, all samples maintain substantial moisture sorption capacity throughout the cycling process. Notably, the peak or equilibrium uptake in each cycle exceed the critical threshold required for the brittle-to-flexible transition. This demonstrates both excellent cyclic stability and strong self-adaptive humidity responsiveness under realistic conditions. Second, cycling behavior was further evaluated under controlled humidity (RH≈68%) using saturated salt solution. The samples underwent 5 sorption-evaporation cycles. In each cycle, samples were exposed for 24 h with periodic mass recording, followed by drying before reuse. As shown in Figure S29c, d, the moisture uptake increases gradually with time during each 24 h cycle and follows similar trends upon repeated cycling. After slight variations in the initial cycles, likely associated with initial structural conditioning, the sorption capacity becomes stable from the third cycle onward, indicating good repeatability and durable sorption- evaporation behavior. Taken together, these results confirm that the aerogels exhibit reliable and reproducible moisture sorption performance under both fluctuating and constant humidity conditions.

**Figure S30.** Comparative mechanical performance of CTS–Cr and CTS–Fe aerogels before and after moisture sorption. (a, d) Uniaxial compression stress–strain curves; (b, e) 100-cycle compression–release curves; (c, f) Three-point bending stress–strain curves. Insets: photos of the corresponding CTS–M aerogels before and after compression or bending.

**Figure S31.** (a) Compressive stress–strain curves of CTS–Fe–D and CTS–Fe–W at 20 °C; (b) Compressive stress–strain curves of CTS–Fe–W at different temperatures; (c, d) Comparison of the compressive stress–strain curves of CTS–Al–W, CTS–Cr–W, and CTS–Fe–W at 60 °C (c) and 80 °C (d), respectively.

**Figure S32.** Moisture uptake of CTS–Al, CTS–Cr, and CTS–Fe aerogels before the temperature-dependent compression tests.

Figure S31 shows the stress–strain curves of CTS–M aerogels at different temperatures. While dry samples (CTS–Fe–D) exhibit abrupt stress drops characteristic of brittle fracture (Figure S31a), moisture-conditioned samples (CTS–Fe–W) display smooth and continuous deformation at 20–40 °C (Figure S31b), indicative of flexible behavior. At higher temperatures (60–80 °C), weak stress fluctuations reappear (Figure S31b), suggesting a partial recovery of brittleness as moisture is reduced. A comparison among CTS–Al–W, CTS–Cr–W, and CTS–Fe–W (Figure S31c–d) reveals that CTS–Al–W maintains smooth deformation even at 80 °C, whereas CTS–Cr–W and CTS–Fe–W show increasing brittle features. This difference correlates well with their initial moisture contents (Figure S32), where CTS–Al–W retains the highest water content. These results indicate that the temperature-dependent mechanical transition is governed by the interplay between thermal desorption and retained moisture. Higher moisture content helps preserve flexibility at elevated temperatures, thereby delaying the brittle transition.

**Figure S33.** Uniaxial compressive modulus of CTS–Fe–W aerogel at different temperatures.

Taking CTS–Fe–W aerogel as a representative example, the modulus increases from 0.16 kPa (20 °C) to 0.39 kPa (40 °C), 0.56 kPa (60 °C), and 1.24 kPa (80 °C) (Figure S33), with a more pronounced rise at elevated temperatures.

**Figure S34.** Moisture sorption of CTS–Al aerogel under ambient dynamic humidity and constant high humidity (RH = 83%).

Under constant high humidity (RH = 83%), CTS–Al aerogel shows an initial increase followed by saturation at ~0.63 g g^−1^ (Figure S34), indicating a finite hygroscopic capacity rather than runaway absorption. Under ambient conditions, the equilibrium uptake remains at a moderate level (~0.45 g g^−1^) and dynamically adjusts with environmental humidity.

**Figure S35.** Photos of CTS–Al–W, CTS–Cr–W, and CTS–Fe–W aerogels before and after manual compression.

To evaluate potential leakage, a direct physical test was performed. The moisture-conditioned samples (CTS–M–W, M=Al, Cr, Fe) were placed on a dry paper and subjected to manual compression. No visible wet marks or liquid traces were observed after removal (Figure S35; Supporting Movie 2). This result indicates that the absorbed water is strongly confined within the internal porous network through hygroscopic interactions and capillary confinement, rather than existing as free liquid. Therefore, the moisture sorption does not lead to leakage during handling or mechanical deformation.

**Figure S36.** 2D SAXS patterns of CTS–M aerogels before (a–c) and after (d–f) moisture sorption.

As shown in the 2D SAXS patterns (Figure S36a–c), the dry-state samples exhibit symmetric concentric scattering rings without any diffraction arcs, indicating isotropic and amorphous internal structures—consistent with the XRD results (Figure S43). Upon moisture sorption (Figure S36d–f), the scattering rings become more diffuse and show a noticeable decrease in intensity, suggesting that water infiltration reduces the electron density contrast between the skeletal framework and the surrounding pore space, thereby weakening overall scattering contrast.

**Figure S37.** SAXS intensity profiles (a) and q-region-dependent slope analysis (b) of CTS–M aerogels before and after moisture sorption.

Further insights are provided by the corresponding 1D SAXS intensity profiles (Figure S37a), which plot scattering intensity as a function of scattering vector q for both dry (D) and hydrated (W) states of CTS–M aerogels. The absence of sharp scattering peaks across all samples reaffirms their amorphous nature, in agreement with the 2D SAXS images. In all cases, the scattering intensity rapidly decreases in the low-*q* region and gradually flattens at higher-*q* region, reflecting a hierarchical porous architecture. Notably, a significant drop in scattering intensity is observed across all hydrated samples, in line with the diffuse ring patterns seen in the 2D SAXS images. This further confirms that water adsorption diminishes internal electron density fluctuations by filling pores, thereby reducing the contrast between different structural domains. To quantitatively evaluate these changes, linear fitting was performed in both the low-*q* and high-*q* regions to extract the scattering slope *k* (Figure S37b). The observed variation in *k* values after hydration implies changes in electron density distribution or interfacial roughness within the aerogel network, reflecting underlying structural rearrangements induced by moisture sorption.

**Figure S38.** Kratky plots (a) and corresponding Guinier-derived radius of gyration (R_g_) values (b) of CTS–M aerogels before and after moisture sorption.

To further elucidate the mesoscale structural evolution of CTS–M aerogels during moisture sorption, the SAXS intensity profiles were weighted by *q*^2^ to generate Kratky plots (Figure S38a). In the dry state, all three samples exhibit rapidly decaying intensities in the low-*q* region without distinct peaks, characteristic of typical amorphous systems lacking long-range order. Upon hydration, the overall scattering intensity further decreases, and the Kratky profiles become notably flatter. This trend suggests that water adsorption weakens inter-chain interactions and reduces local structural order within the network. To quantitatively assess these changes, Guinier fitting was performed in the low-*q* region of the Kratky plots, and the radius of gyration (*R*_g_) was calculated according to the Guinier approximation (Equation 7, Figure S38b):$I\left(q\right)=I\left(0\right)\mathrm{e}^{-q^{2}R_{g}^{2}/3}$ (7)

where *q* is the scattering vector, *I* (0) is the intensity at zero angle, *I* (*q*) is the scattering intensity, and *R*_g_ is the radius of gyration.

The results reveal that *R*_g_ remains nearly unchanged before and after moisture sorption, indicating that the hydration process induces only localized chain relaxation or pore-wall swelling, rather than large-scale dimensional expansion. This finding is consistent with SEM observations of reduced pore number and increased wall thickness (Figure 1h–j), as well as the negligible change in macroscopic geometry observed in optical images of the hydrated gels (Figure S22d).

**Figure S39.** Porod’s law deviation and correction curves of aerogels before and after moisture sorption. (a) CTS–Cr, (b) CTS–Fe.

Figure S39 shows the Porod-deviation corrected plots of CTS–M aerogels in both dry and wet states, aiming to evaluate the interfacial structural evolution upon moisture sorption. According to Porod’s law, an ideal two-phase system with sharp interfaces follows the power-law relation (Equations 8 or 9), where the Porod constant *K_P_* is proportional to the total surface area of the scattering entities. Any deviation from this behavior in the high-*q* region indicates non-ideal interfacial characteristics.

$\lim_{q\to\infty} I\left(q\right)q^{4}=K_{P}$ (8)

$\lim_{q\to\infty} \ln\left[I\left(q\right)q^{4}\right]=\ln K_{P}$ (9)

As shown in Figure S39a, b, all samples exhibit noticeable upward curvature in the high-*q* region, indicating typical positive Porod deviation, which implies the presence of electron density fluctuations within the scattering phase—commonly observed in amorphous porous materials. In the dry state, this deviation is already evident, suggesting inherent internal density heterogeneity within the aerogel skeleton. After moisture sorption, the degree of positive deviation is universally reduced, as manifested by the downward shift of the fitted curves. This attenuation indicates that the introduction of water decreases the electron density contrast between the framework and pore regions, thereby reducing the interfacial gradient and promoting smoother transitions across phase boundaries. This trend highlights a universal structural homogenization pathway during hydration. Moreover, the reduction in the Porod constant *K* after moisture sorption reflects a decrease in the overall specific surface area, in agreement with SEM observations (Figure 1i, j) and BET results (Table S3), which together confirm that water infiltration effectively fills pores and diminishes accessible internal surface area.

**Figure S40.** Double-log plots (a, b) and fractal dimension fitting results (c, d) of CTS–M aerogels before and after moisture sorption.

Fractal dimension is a key quantitative parameter used to characterize the structural roughness (surface fractal) or compactness (mass fractal) of aerogel skeletons across different length scales, providing valuable insights into structural complexity changes during the moisture sorption process. Based on the power-law relationship (Equation 10), the fractal dimension of CTS–M aerogels was determined by linear fitting of the log–log plot of ln *I*(*q*) versus ln *q*, and further calculated using Equation 11.

$I\left(q\right)=I_{0}q^{-\alpha}$ (10)

$\ln I\left(q\right)=\ln I_{0}-\alpha\ln q$ (11)

where *α* represents the Porod slope, which directly determines the type and magnitude of the fractal dimension: 0≤*α*≤3, the system exhibits mass fractal behavior with *D*_m_=*α*; when 3<α≤4, it indicates surface fractal characteristics, with *D*_s_=6–α.

As shown in Figure S40a, b, the log–log scattering curves of all samples exhibit distinct linear regimes in the low- to mid-q range with excellent fitting quality (*R*^2^>0.99), confirming the existence of fractal structural features. Further fitting results (Figure S40c, d and Table S4) indicate that CTS–Cr displays surface fractal behavior, while CTS–Fe shows mass fractal characteristics. This difference can be corroborated by SEM images (Figure 1f, g): the CTS–Cr aerogel exhibits well-defined framework boundaries, abundant pores, and a rough surface, where the scattering is dominated by interfacial contrasts, consistent with surface fractality; in contrast, CTS–Fe features a denser and smoother structure with less distinguishable pore interfaces, suggesting that the scattering arises mainly from internal density fluctuations, consistent with mass fractality. Notably, the fractal dimensions of both aerogels decrease after moisture sorption (from 2.7 to 2.4 for Cr, and from 2.5 to 2.3 for Fe), indicating a reduction in surface roughness or internal structural complexity and a structural evolution toward a more regular and homogeneous state. This trend is consistent with SEM observations (Figure 1i, j), which show pore shrinkage, skeleton swelling, and surface smoothing. These results collectively demonstrate that water molecules effectively modulate the density contrast and spatial configuration between the skeleton and pores at the microscale, thereby promoting structural homogenization across multiple length scales.

**Figure S41.** Pair distance distribution function (PDDF) curves of CTS–Cr (a) and CTS–Fe (b) aerogels before and after moisture sorption.

Based on the SAXS intensity data, the pair distance distribution function *P*(*r*) was obtained via indirect Fourier transformation, as defined in Equation (12), to investigate the real-space structural evolution of the aerogels during moisture sorption. This function describes the probability distribution of intraparticle distances *r*, serving as a crucial bridge between scattering intensity and spatial structure, with its mathematical formulation shown in Equation (13).

$P\left(r\right)=\frac{r^{2}}{2\pi^{2}}\int_{0}^{\infty} q^{2}I\left(q\right)\frac{\sin\left(qr\right)}{qr}dq$ (12)

$I\left(q\right)=4\pi\int_{0}^{D_{\max}} P\left(r\right)\frac{\sin\left(qr\right)}{qr}dr$ (13)

As illustrated in Figure S41, both CTS–Cr and CTS–Fe aerogels exhibit a noticeable reduction in PDDF peak intensity and a narrowing of the distribution width after moisture sorption. These changes indicate that water molecules infiltrating the porous skeleton suppress long-range density fluctuations and reduce the characteristic scattering length scale. This observation provides complementary real-space evidence supporting the moisture-induced structural densification and homogenization trends revealed by SAXS-based multiscale analysis.

**Figure S42.** Nitrogen adsorption–desorption isotherms (a) and pore size distribution curves (b, c) of CTS–Al aerogels before and after moisture sorption.

To further elucidate the pore structure evolution during moisture sorption, nitrogen adsorption–desorption measurements were conducted to evaluate changes in the microporous and mesoporous regimes of CTS–Al aerogels (Figure S42). SEM images previously confirmed that the aerogel exhibits a macropore-dominated structure, and post-absorption morphological features such as pore filling and wall swelling were clearly observed. Therefore, mercury intrusion method was not employed, and the analysis was focused on the micro/mesoporous range. Due to extremely low adsorption capacities and unstable isotherms for CTS–Cr and CTS–Fe aerogels, only CTS–Al was selected for representative analysis. As shown in Figure S42a, the dry-state CTS–Al aerogel exhibited a typical type IV isotherm with an H3 hysteresis loop, indicating mesoporous characteristics. After moisture sorption, the nitrogen adsorption amount dropped nearly to zero, with BET surface area plummeting from 9.7 m^2^ g^−1^ to 0.5 m^2^ g^−1^ and total pore volume decreasing from 0.0202 cm^3^ g^−1^ to 0.0007 cm^3^ g^−1^ (Table S3). These results confirm that a significant fraction of the micro/mesopores became occupied by water molecules, blocking access to nitrogen during measurement. The BJH pore size distributions (Figure S42b, c) further revealed that the initial pores were concentrated in the 3–5 nm mesoporous region, which nearly vanished after hydration, indicating a homogenized and densified pore structure. This analysis at the micro/mesoscale level further validates the hydration-induced pore densification trend and supports the concept of progressive water infiltration into multiscale channels. Although the overall BET surface area is relatively low and micro/mesopores contribute minimally to total water uptake, they serve as crucial initial adsorption and retention sites. These sites facilitate localized wetting and progressive structural reorganization, thereby providing a nanoscale foundation for chain segment relaxation and macroscopic framework densification.

**Figure S43.** XRD patterns of CTS–Al (a), CTS–Cr (b), and CTS–Fe (c) aerogels before and after moisture sorption.

XRD patterns (Figure S43) further corroborate the structural stability, showing broad and weak amorphous humps in both dry and hydrated samples. The absence of any newly formed diffraction peaks or noticeable peak shifts confirms that water sorption does not induce any crystallization or long-range structural transitions. The reduced peak intensity observed after hydration implies disruption of short-range ordering within the polymer network. This effect likely arises from three concurrent factors: (i) partial hydration of the metal ions, which perturbs the original metal–chitosan coordination environment; (ii) formation of hydrogen bonds between water molecules and chitosan’s amino and hydroxyl groups, weakening intermolecular interactions and causing chain relaxation; and (iii) filling of pores by water molecules, which homogenizes the local electron density and decreases overall diffraction contrast. In summary, CTS–M aerogels exhibit excellent chemical and structural stability during the moisture sorption process. The moisture-induced softening behavior is primarily driven by physical effects such as structural swelling and weakened intermolecular interactions, rather than by any inherent chemical degradation of the polymer framework.

**Figure S44.** XPS spectra comparison of CTS–M aerogels before and after moisture sorption. (a) CTS–Al, (b) CTS–Cr, (c) CTS–Fe.

The survey spectra (Figure S44) show clear signals for C 1s, O 1s, N 1s, and the respective metal ions (Al 2p, Cr 2p, Fe 2p) in both dry and wet states, indicating successful complexation between trivalent metal chlorides and chitosan, and confirming that the basic chemical framework remains intact during moisture exposure. Notably, a general decrease in signal intensity is observed after hydration, likely due to structural swelling that increases the electron escape depth and thus attenuates surface-sensitive XPS signals.

**Figure S45.** High-resolution XPS spectra comparison of CTS–Al aerogel before and after moisture sorption. (a, b) Al 2p, (c, d) C 1s, (e, f) O 1s, and (g, h) N 1s.

**Figure S46.** High-resolution XPS spectra comparison of CTS–Cr aerogel before and after moisture sorption. (a, b) Cr 2p, (c, d) C 1s, (e, f) O 1s, and (g, h) N 1s.

**Figure S47.** High-resolution XPS spectra comparison of CTS–Fe aerogel before and after moisture sorption. (a, b) Fe 2p, (c, d) C 1s, (e, f) O 1s, and (g, h) N 1s.

High-resolution metal spectra (Figures S45a, b; S46a, b; and S47a, b) show negligible changes in binding energy and peak shape, indicating that both the oxidation state and the coordination environment of the M^3+^ ions remain stable after moisture sorption. Similarly, the C 1s spectra (Figures S45c, d; S46c, d; and S47c, d) display consistent peak positions and relative intensities for C−C, C−O, and C=O species, confirming that the carbon backbone remains chemically intact and no bond cleavage occurs. The O 1s and N 1s spectra (Figures S45e–h; S46e–h; and S47e–h) also exhibit minimal shifts in peak positions and areas, which may be attributed to subtle local changes in the electronic environment caused by hydrogen bond reorganization—a mechanism consistent with the FTIR-based "hydrogen bond reconstruction" hypothesis (Figure S23a–c).

**Figure S48.** Infrared thermal images and 3D temperature distribution of CTS–M aerogels on a 110°C hot plate. (a, d) CTS–Al, (b, e) CTS–Cr, (c, f) CTS–V.

**Figure S49.** Infrared thermal images and 3D temperature distribution of CTS–M aerogels on −196°C cold plate. (a, d) CTS–Al, (b, e) CTS–Cr, (c, f) CTS–V.

**Figure S50.** Comparison of GCD curves at a current density of 0.5 A g^−1^ between C and C–V_2_O_3_ aerogels.

**Figure S51.** GCD curves of C–V_2_O_3_ aerogels prepared from CTS precursors with different viscosities.

Among the three variants, the sample synthesized from medium-viscosity CTS (20-100 mPa·s) delivers the longest discharge plateau, and the most symmetric galvanostatic curves (Figure S51), indicative of superior specific capacity and energy-output stability.

**Figure S52.** SEM images and corresponding elemental mapping of C–V_2_O_3_–G aerogels prepared from CTS precursors with different viscosities. (a–c) 5-20 mPa·s, (d–f) 20-100 mPa·s, (g–i) 200-600 mPa·s.

The performance disparity is rationalized by analysing the structural evolution. SEM images at different magnifications (Figure S52) show that low-viscosity CTS (5-20 mPa·s; Figure S52a–c) yields loosely connected, polydisperse nanoparticles within an incoherent 3D network, whereas high-viscosity CTS (200-600 mPa·s; Figure S52g–i) produces pronounced particle agglomeration and partial pore collapse. In contrast, the medium-viscosity CTS (20-100 mPa·s; Figure S52d–f) forms a monodisperse, nano-crystalline, sphere-like scaffold endowed with well-interconnected hierarchical pores, markedly facilitating electron transport and ion diffusion.

**Figure S53.** Raman spectra of C–V_2_O_3_ aerogels. (a) Samples derived from CTS precursors with different viscosities, (b) Samples carbonized at different temperatures.

Raman spectroscopy (Figure S53a) shows *I*_D_/*I*_G_ values of 0.94, 0.95, and 0.89 for the low-, medium-, and high-viscosity samples, respectively, with the medium-viscosity sample exhibiting the highest degree of structural disorder. Such moderate disorder introduces abundant edge-defect sites, enhancing pseudocapacitive contributions and establishing a clear structure-property correlation with the observed high energy-storage capability. These structural and electrochemical differences originate from viscosity-dependent coordination of chitosan, which is intrinsically linked to its degree of deacetylation (DDA). Increasing viscosity generally accompanies higher DDA, elevating the density of −NH_2_ groups along the polymer backbone and thus strengthening coordination with V^3+^ ions. Low-DDA systems lack sufficient –NH_2_ content, resulting in poor complexation efficiency and an unstable framework, whereas excessive −NH_2_ groups in high-DDA systems induce intermolecular entanglement and particle aggregation, compromising structural integrity and homogeneity. The medium-DDA system (~80%) achieves an optimal balance between coordination uniformity and framework robustness, yielding a structurally uniform, electrically percolating, and defect-rich C–V_2_O_3_ aerogel that underpins the markedly enhanced electrochemical performance. Raman spectra (Figure S53b) exhibit the characteristic D (1336 cm^−1^) and G (1585 cm^−1^) bands for all temperatures, with *I*_D_/*I*_G_ ratios clustering between 0.92 and 0.96, confirming that a well-developed, structurally robust carbon framework is established above 700 °C. Intriguingly, the 900 °C material registers the highest *I*_D_/*I*_G_ value, indicating greater disorder; this correlates with the best electrochemical outcome, underscoring the beneficial role of defect-rich carbon in promoting ion adsorption and charge transport.

**Figure S54.** Electrochemical performance of C–V_2_O_3_ aerogels prepared from different colored precursors. (a) GCD curves at 0.5 A g^−1^, (b) Nyquist plots.

GCD profiles of C–V_2_O_3_–G delivers the longest discharge plateau and the highest specific capacity (Figure S54a). Electrochemical-impedance spectroscopy (Figure S54b) further confirms that C–V_2_O_3_–G exhibits the smallest semicircle diameter and the steepest low-frequency Warburg slope, corresponding to the lowest charge-transfer resistance (R_ct_) and diffusion impedance.

**Figure S55.** Photos of an electrolyte droplet of 2 M ZnSO_4_ on the surface of the C–V_2_O_3_ aerogel electrode.

**Figure S56.** SEM images and corresponding elemental mapping of C–V_2_O_3_ aerogels with different colors. (a–d) C–V_2_O_3_–T, (e–h) C–V_2_O_3_–G, (i–l) C–V_2_O_3_–B. Please note that the sample in Figure S56a–d is the same as in Figures 1l, m and 2e.

After carbonization, the resulting C–V_2_O_3_–T, C–V_2_O_3_–G, and C–V_2_O_3_–B samples preserved their respective structural frameworks, while their surfaces developed dense nanoparticle coatings, forming core–shell-like morphologies (Figure S56). Although all three aerogels are uniformly sheathed by V_2_O_3_ nanocrystallites, C–V_2_O_3_–T retains a loosely connected fibrous backbone, whereas C–V_2_O_3_–G and C–V_2_O_3_–B evolve into compact, sesame-ball-like architectures in which the closely packed secondary spheres create a well-interlinked hierarchical pore network favourable for electron/ion transport.

XRD patterns (Figure S57a) display the characteristic reflections of rhombohedral V_2_O_3_ (JCPDS 34-0187) together with the broad hump of amorphous carbon (~2θ = 20°); among them, C–V_2_O_3_–G presents the sharpest and most intense diffraction peaks, indicative of superior crystallinity. Weak VN reflections at 2θ = 37.6° and 43.7° (JCPDS 35-0768) are discernible for C–V_2_O_3_–T and C–V_2_O_3_–G, suggesting a local nitridation reaction derived from the chitosan backbone or the N₂ atmosphere. The trace VN phase is expected to enhance electronic conductivity. XRD patterns (Figure S57b) show that all specimens retain the rhombohedral V_2_O_3_ phase (JCPDS 34-0187) together with the broad (002) band of amorphous carbon, indicating an invariant global framework. With increasing carbonisation temperature, the V_2_O_3_ reflections sharpen progressively; they reach maximum intensity at 900 °C, evidencing optimal crystallinity. Notably, weak VN reflections emerge first at 900 °C and intensify at 1000 °C, where the V_2_O_3_ peaks concomitantly attenuate, implying a significant phase conversion. Although a trace amount of VN can enhance electronic conduction, excessive nitridation is liable to attenuate the intimate carbon/V₂O₃ interface and reduce the density of redox-active sites, thereby compromising capacity. The synergistic coexistence of highly crystalline V₂O₃ with a minor VN fraction in the 900 °C sample is therefore considered pivotal for its superior performance.

**Figure S57.** Comparative XRD patterns of C–V_2_O_3_ aerogels. (a) C–V_2_O_3_ aerogels derived from different-color precursors, (b) C–V_2_O_3_–G aerogels prepared under different carbonization temperatures.

**Figure S58.** Electrochemical performance of C–V_2_O_3_–G aerogels at different carbonization temperatures. (a) CV curves at 5 mV s^−1^, (b) Nyquist plots.

The CV traces (Figure S58a) further display the most symmetrical redox couples and the largest integrated area, reflecting faster kinetics and larger charge-storage capability, while EIS spectra (Figure S58b) reveal the lowest charge-transfer resistance, attesting to superior electronic conductivity.

**Figure S59.** SEM images and corresponding elemental mapping of C–V_2_O_3_–G aerogels prepared at different carbonization temperatures. (a–d) 700°C, (e–h) 800°C, (i–l) 900°C.

**Figure S60.** Rate performance of the optimized C–V_2_O_3_–G–900 electrode in aqueous Zn//2 M ZnSO_4_//C–V_2_O_3_ ZIBs.

The sample achieves a high specific capacity of 341 mAh g^−1^ at 0.1 A g^−1^, retaining 89 mAh g^−1^ at 10 A g^−1^ (Figure S60), with Coulombic efficiency of 85–100%, confirming excellent electrochemical reversibility and structural stability.

**Figure S61.** Capacitive contribution (blue-shaded areas) of the C–V_2_O_3_–G–900 electrode at scan rates of 1 mV s^−1^ (a), 2 mV s^−1^ (b), 5 mV s^−1^ (c), 10 mV s^−1^ (d), 15 mV s^−1^ (e), and 20 mV s^−1^ (f).

The spatial distribution of the capacitive current, visualised by the shaded regions in Figure S61a–f, expands conspicuously with increasing scan rate, further substantiating the dynamic shift toward pseudocapacitive charge storage during rapid charge–discharge operation.

**Figure S62.** Ex-situ XRD patterns of the C–V_2_O_3_ electrode at different charge/discharge states. (a) XRD patterns of the pristine electrode and electrodes collected at selected charge/discharge states after 20 CV activation cycles (D and C denote discharge and charge states, respectively). (b) Enlarged XRD patterns of the V_2_O_3_ (122) peak at representative redox states. (c) XRD patterns of electrodes charged to 1.0 V after 20 and 50 cycles.

To minimize the interference from the initial activation, the electrode was first subjected for 20 CV cycles at 5 mV s⁻^1^ until stable CV profiles were obtained. The ex-situ XRD sampling potentials were then selected based on the main redox peaks in the CV curves and the charge/discharge plateaus in the GCD profiles (0.1 A g⁻^1^), including representative discharge (D–0.6 V, D–0.9 V, D–1.0 V) and charge (C–0.7 V, C–1.0 V, C–1.6 V) states. Pristine electrode and graphite paper were also measured as references to exclude contributions from the current collector and electrolyte residues.

The ex-situ XRD results (Figure S62a) show that the characteristic diffraction peaks of V_2_O_3_ (e.g., 33°, 39°, 41° and 58°) remain clearly identifiable at all states, indicating that the V_2_O_3_ host structure is well preserved during Zn^2+^ insertion/extraction. Additional low-angle peaks observed in discharged states are attributed to residual electrolyte species (e.g., ZnSO_4_·6H_2_O) rather than structural degradation. Notably, the V_2_O_3_ (122) peak exhibits a reversible shift (Figure S62b)—moving to lower 2θ during discharge and returning to higher 2θ values upon charging—indicating reversible lattice expansion/contraction associated with Zn^2+^ insertion/extraction, consistent with previous reports^[5]^. Furthermore, the electrode charged to 1.0 V after 50 cycles exhibits an XRD pattern highly similar to that after 20 cycles (Figure S62c), confirming the reproducibility of the structural evolution upon prolonged cycling. These results demonstrate that the C–V_2_O_3_ cathode undergoes reversible lattice expansion and contraction while preserving the integrity of the V_2_O_3_ host framework. This structural reversibility provides direct evidence for the observed cycling stability and electrochemical reversibility.

**Figure S63.** Ex-situ Raman spectra of the C–V_2_O_3_ electrode collected at different charge/discharge states.

The ex-situ Raman spectra (Figure S63) provide complementary insight into the stability of the carbon framework. All samples retain the characteristic D and G bands of carbon, with I_D_/I_G_ values varying only slightly within the range of 0.97–1.06. This minimal variation indicates that the conductive carbon network remains structurally stable throughout cycling, without significant irreversible degradation.

**Figure S64.** Cycling performance and Coulombic efficiency of the C–V_2_O_3_–G–900 electrode over 10000 cycles at 5 A g^−1^.

**Figure S65.** SEM images of the electrodes in Zn//2M ZnSO_4_//C–V_2_O_3_ ZIBs before and after 120,000 cycles. (a–c) Pristine cathode (a, b) and anode (c); (d–f) cycled cathode (d, e) and anode (f).

The morphologies of both cathode and anode were compared before and after cycling (Figure S65). Prior to testing (Figure S65a, b), the C–V_2_O_3_ cathode exhibits uniformly dispersed, densely packed spherical particles with sharp interfaces. After 120,000 cycles (Figure S65d, e), the overall spherical framework is largely preserved, although inter-particle contacts become slightly loosened and the spheres show modest surface roughening and size diminution—evidence that the robust scaffold maintains continuous electronic pathways during prolonged operation, underpinning the outstanding cycling stability. In contrast, the Zn anode undergoes pronounced irreversible changes. The pristine foil (Figure S65c) presents a smooth surface with visible machining marks, indicating a clean interface; after cycling (Figure S65f), it is covered by extensive flake-like crystalline deposits, signalling non-uniform Zn^2+^ plating/stripping and side reactions. Such evolution promotes dendritic growth, interfacial polarisation, and localised stress, which collectively account for the early-stage capacity fade.

**Figure S66.** GCD curves at 0.1 A g^−1^ for the first three cycles of the all-solid-state Zn//gelatin//C–V_2_O_3_ ZIBs, inset: corresponding voltage–time profiles.

**Figure S67.** Nyquist plot comparison between aqueous and all-solid-state Zn//C–V_2_O_3_ ZIBs.

Figure S67 contrasts the impedance characteristics of the C–V_2_O_3_ aerogel in liquid-electrolyte and all-solid-state zinc-ion cells, thereby elucidating the influence of electrolyte configuration on interfacial kinetics. As shown in Figure S67, both Nyquist plots consist of a high-frequency semicircle and a low-frequency inclined line, assigned to the charge-transfer resistance (R_ct_) and the Warburg diffusion impedance (Z_w_), respectively; the intercept on the real axis denotes the ohmic resistance (R_s_). Relative to the aqueous cell, the gel-electrolyte device exhibits a larger semicircle, a steeper low-frequency slope, and a higher real-axis intercept, indicating markedly increased R_ct_ and R_s_—attributable to restricted ionic mobility within the gelatin matrix and elevated interfacial contact resistance. Despite the higher overall impedance, the well-defined, distortion-free profile of the solid-state spectrum attests to interfacial stability and a coherent electrochemical response. Equivalent-circuit fitting (Figure 5d) reproduces the impedance spectra with a simple R_s_–R_ct_–Z_w_ series model, yielding excellent agreement and confirming the absence of short-circuit pathways or interfacial mismatches. The corresponding Bode plot (Figure 5d) further delineates the frequency response of the solid-state cell: in the high-frequency region, the phase angle exhibits a characteristic lag typical of capacitive interfacial behaviour, while across the entire frequency range only a single phase-angle maximum is observed. This single time constant mirrors the solitary semicircle in the Nyquist plot, indicating that charge transport is dominated by one well-defined kinetic process. In sum, although the all-solid-state configuration incurs higher charge-transfer resistance, it maintains a stable interface with a clear, analy­sable frequency response, underscoring its electrochemical coherence and controllability.

**Figure S68.** Electrochemical performance of the C–V_2_O_3_–G aerogels cathode in all-solid-state ZIBs. (a) CV curves at different scan rates, (b) log(i) versus log(v) plots at the peak current, (c) Capacitive contribution (cyan-shaded areas) at scan rates of 5 mV s^−1^, and (d) Contribution ratios of capacitive and diffusion-controlled capacities at various scan rates.

To elucidate the charge‐storage mechanism of the C–V_2_O_3_ cathode in the all-solid-state configuration, Figure S68 examines its rate-dependent electrochemical response and the relative contributions of diffusion-limited and surface-capacitive processes. As illustrated in Figure S68a, cyclic-voltammetry profiles collected between 1 and 20 mV s⁻^1^ retain well-defined redox couples; the peak currents rise proportionally with scan rate while the overall curve morphology remains unaltered, evidencing excellent reversibility and rate adaptability. Log–log plots of peak current versus scan rate (Figure S68b) yield *b*-values of 0.67 (charge) and 0.70 (discharge), signifying a mixed charge-storage regime in which semi-infinite diffusion and pseudocapacitive surface reactions coexist. Quantitative partitioning of these two contributions was carried out using the established current–scan-rate relation 𝑖(𝑉)=𝑘_1_𝑣+𝑘_2_𝑣^1/2^. The capacitive fraction, visualised as the cyan-shaded area in the CV curve recorded at 5 mV s⁻^1^ (Figure S68c), accounts for 27.7%, indicating that diffusion-controlled intercalation dominates at low scan rates. When the scan rate is increased from 1 to 20 mV s^−1^ (Figure S68d), the capacitive contribution rises markedly—from 16.1% to 49.5%—demonstrating that surface‐controlled kinetics progressively prevail under high-rate operation, thereby enhancing charge-transfer efficiency. Collectively, these results confirm that the C–V_2_O_3_ aerogel operates via a hybrid storage mechanism, with the balance between diffusion-limited and capacitive processes tunable by the scan rate. This synergistic behaviour underpins the electrode’s ability to deliver both high capacity at low rates and excellent power performance at elevated rates, making it well positioned for high-performance all-solid-state zinc-ion energy-storage applications.

**Figure S69.** Cycling stability and Coulombic efficiency of quasi-solid-state Zn//gelatin//C–V_2_O_3_ ZIBs over 10000 cycles at 5 A g⁻^1^.

**Table S1.** Density and thermal conductivity of CTS–M aerogels

| **Sample** | **Density (g cm**^–^**^3^)** | **Thermal conductivity (W m**^−1^ **K**^−1^**)** |
| --- | --- | --- |
| CTS–Al | 0.068 | 0.061 |
| CTS–Cr | 0.071 | 0.051 |
| CTS–Fe | 0.073 | 0.052 |

**Table S2.** Pore structure parameters of CTS–V precursors with different colors and their carbonization products C–V_2_O_3_

| **Sample** | **Surface Area**  **(m^2^ g**^−1^**)** | **Pore Volume**  **(cm^3^ g**^−1^**)** | **Average Pore Diameter**  **(nm)** |
| --- | --- | --- | --- |
| CTS–V–T | 27 | 0.019 | 3.8 |
| CTS–V–G | 20 | 0.021 | 3.4 |
| CTS–V–B | 24 | 0.017 | 3.4 |
| C–V_2_O_3_–T–900 | 438 | 0.118 | 3.8 |
| C–V_2_O_3_–G–900 | 457 | 0.094 | 3.8 |
| C–V_2_O_3_–B–900 | 448 | 0.108 | 3.8 |

Before carbonization, all CTS–V precursors exhibit low BET surface areas (ranging from 20 to 27 m^2^ g^−1^) and pore volumes (0.017 and 0.021 cm^3^ g^−1^). After carbonization, the surface area dramatically increases to 438–457 m^2^ g^−1^, accompanied by a pore volume increase to 0.094–0.118 cm^3^ g^−1^ and a nearly unchanged average pore diameter of ~ 3.8 nm, indicating effective pore reconstruction without collapse or over-shrinkage. Notably, the C–V_2_O_3_–G sample shows the highest surface area (457 m^2^ g^−1^), despite originating from a precursor with the lowest initial value (20 m^2^ g^−1^), suggesting more extensive micropore/mesopore development and skeletal reorganization during pyrolysis. This enhanced porosity and network connectivity are beneficial for electrolyte wetting and ion diffusion, correlating well with its superior electrochemical performance observed in Figure 4d. In summary, BET analysis provides quantitative insights into pore evolution from precursor to carbonized product, complementing the morphological observations from SEM and offering a structural basis for understanding the materials’ electrochemical behavior.

**Table S3.** Pore structure parameters of CTS–Al aerogels before and after moisture sorption.

| **Sample** | **Surface Area**  **(m^2^ g**^−1^**)** | **Pore Volume**  **(cm^3^ g**^−1^**)** | **Average Pore Diameter**  **(nm)** |
| --- | --- | --- | --- |
| CTS–Al–D | 9.7 | 0.020 | 7.4 |
| CTS–Al–W | 0.5 | 0.001 | 7.8 |

**Table S4.** Fitting results of fractal dimension parameters for CTS–M aerogels before and after moisture sorption.

| **Sample** | **α** | **D** | **slope** | **R^2^** |
| --- | --- | --- | --- | --- |
| CTS–Cr–D | 3.3 | D_s_=2.7 | -3.28513 | 0.99921 |
| CTS–Cr–W | 3.6 | D_s_=2.4 | -3.60285 | 0.99779 |
| CTS–Fe–D | 2.5 | D_m_=2.5 | -2.47443 | 0.99924 |
| CTS–Fe–W | 2.3 | D_m_=2.3 | -2.25528 | 0.99606 |

**Table S5.** Comparison of electrochemical properties of different vanadium oxides composites and carbon materials applied to aqueous ZIBs (zinc foil as anode)

| **Cathode** | **Voltage range (V)** | **Current density (A g^−1^)/ Specific capacity (mAh g^−1^)** | **Current density**  **(A g^−1^)/ Cycle num**b**er/ Capacity retention** | **Electrolyte** |
| --- | --- | --- | --- | --- |
| **C@V_2_O_3_ aerogel**  **(This work)** | **0-2** | **0.1/341** | **5/10000/85%**  **5/120000/50%** | **2M ZnSO_4_** |
| p-V_2_O_3_@C ^[20]^ | 0.3-1.5 | 0.1/300 | 5/4000/90% | 3M Zn(CF_3_SO_3_)_2_ |
| V_2_O_3_@C ^[7]^ | 0.2-1.6 | 0.5/325.7 | 10/10000/126% | 2M Zn(CF_3_SO_3_)_2_ |
| V_2_O_3_@C ^[21]^ | 0.2-1.6 | 0.5/389 | 5/2000/78% | 3M Zn(CF_3_SO_3_)_2_ |
| V_2_O_3_@CNF ^[22]^ | 0.2-1.6 | 0.1/336.2 | 12/2000/99.7% | 3M Zn(CF_3_SO_3_)_2_ |
| V_2_O_3_/NC ^[23]^ | 0.2-1.6 | 0.5/405 | 20/4000/90.6% | 3M Zn(CF_3_SO_3_)_2_ |
| V_2_O_3_@C ^[24]^ | 0.2-1.6 | 1/506.1 | 12/10000/90.3% | 3M Zn(CF_3_SO_3_)_2_ |
| VO_2_@NC ^[25]^ | 0.2-1.4 | 0.5/495.2 | 10/2500/99.7% | 3M Zn(CF_3_SO_3_)_2_ |
| C@VO_2_ ^[26]^ | 0.2-1.4 | 0.5/324.2 | 5/600/84.3% | 3 M Zn(OTf)_2_ |
| C@V_2_O_5_ ^[27]^ | 0.2-1.6 | 0.5/361.7 | 0.5/2000/71% | 2M Zn(CF_3_SO_3_)_2_ |
| V_2_O_5_@CNTs ^[28]^ | 0.2-1.6 | 1/418.0 | 5/2500/91.9% | 3M Zn(CF_3_SO_3_)_2_ |
| (NH_4_)_2_V_3_O_8_/C ^[29]^ | 0.4-1.6 | 0.1/356 | 1/2000/50.8% | 3M Zn(CF_3_SO_3_)_2_ |
| Na_3_V_2_(PO_4_)_3_/rGO ^[30]^ | 0.6-1.8 | 0.05/107 | 0.5/200/75% | 2M Zn(CF_3_SO_3_)_2_ |
| V_2_O_3_ ^[5]^ | 0.1-1.3 | 0.1/196 | 5/30000/81% | 3M Zn(CF_3_SO_3_)_2_ |
| V_2_O_3_ (commercial) ^[22]^ | 0.2-1.6 | 0.1/233.1 |  | 3M Zn(CF_3_SO_3_)_2_ |
| V_2_O_3_ ^[31]^ | 0.2-1.6 | 0.8/328 | 3.2/800/97.3% | 3M Zn(CF_3_SO_3_)_2_ |
| VO_2_(B) ^[32]^ | 0.3-1.8 | 0.1/330 | 0.5/100/65% | 3M Zn(CF_3_SO_3_)_2_ |
| V_2_O_5_ nanofibers ^[33]^ | 0.5-1.5 | 3/104 | 0.6/500/81% | 3M Zn(CF_3_SO_3_)_2_ |
| V_3_O_7_·H_2_O ^[34]^ | 0.4-1.1 | 0.375/375 | 3/200/80% | 1 M ZnSO_4_ |
| CuVO ^[35]^ | 0.3-1.4 | 1/285 | 10/10000/88% | 2M ZnSO_4_ |
| HVO ^[36]^ | 0.4-1.4 | 0.1/323 | 10/1000/64% | 3 M ZnSO_4_ |
| AC (commercial) ^[37]^ | 0.2-1.8 | 0.1/121 | 1/10000/91% | 2M ZnSO_4_ |
| MCHSs ^[38]^ | 0.2-1.8 | 0.1/174 | 1/10000/96% | 2M ZnSO_4_ |
| HBC ^[39]^ | 0.2-1.8 | 0.1/95.4 | 1/1000/＞100% | 2M ZnSO_4_ |
| NPCN ^[40]^ | 0.1-1.8 | 0.1/204.7 | 5/20000/95.5% | 1M Zn(CF_3_SO_3_)_2_ |
| CNPK ^[41]^ | 0.2-1.8 | 0.1/103 | 5/10000/101.8% | 1M ZnSO_4_ |
| GH ^[42]^ | 0.2-1.8 | 0.2/99.3 | 15/10000/90% | 2M ZnSO_4_ |
| TAP/Ti_3_C_2_T_x_ ^[43]^ | 0.2-1.5 | 0.1/267 | 1/10000/81.6% | 2M ZnSO_4_ |

To benchmark the overall performance of the present C–V_2_O_3_ aerogel, Table S5 compiles the key electrochemical metrics of representative vanadium oxides (e.g., V_2_O_3_ ^[5, 22, 31]^, VO_2_ ^[32]^, V_2_O_5_ ^[33]^, V_3_O_7_ ^[34]^), carbonaceous materials (commercial activated carbon ^[37]^, porous carbon ^[38-41]^, graphene-based materials ^[42]^), and vanadium-oxide/carbon composites (V_2_O_3_@C ^[7, 20-24]^, VO_2_@C ^[25, 26]^, V_2_O_5_@C ^[27, 28]^, (NH_4_)_2_V_3_O_8_/C ^[29]^) recently reported for aqueous Zn-ion batteries. In terms of operating window, the C–V_2_O_3_ aerogel delivers an exceptionally wide potential range of up to 2.0 V—markedly higher than that of state-of-the-art V_2_O_3_ electrodes (1.2–1.4 V), stand-alone carbon materials (1.6–1.7 V), V_2_O_3_/C composites (1.2–1.4 V), other vanadium oxides V_x_O_y_(0.7–1.5 V) or their carbon hybrids (1.2–1.4 V), and even exceeding several systems employing highly concentrated electrolytes (e.g. 3 M Zn(CF_3_SO_3_)_2_). With respect to durability, although the capacity retention after 10 000 cycles (85 %) is marginally lower than a few literature benchmarks (> 90 %), the electrode excels in long-term resilience: at 5 A g^−1^ it sustains 120 000 cycles with 50 % capacity retention—performance that remains rare for aqueous Zn-ion systems. Moreover, the electrode affords a high specific capacity of 327.8 mAh g**^−1^** at 0.1 A g**^−1^**, which—while not the global record—surpasses the majority of reported counterparts. Collectively, the C–V_2_O_3_ aerogel achieves an attractive balance among voltage window, capacity, and ultralong cycling stability, highlighting the effectiveness of the structure–composition co-engineering strategy adopted herein.

A comprehensive comparison with state-of-the-art solid-state Zn-ion batteries is summarised in Table S6. Relative to various vanadium oxides, pure carbon materials, and carbon/oxide composites, the C–V_2_O_3_-based device affords an unprecedentedly wide voltage window of 2.0 V (other systems typically 0.8–1.7 V) ^[28, 43-49]^. Moreover, it retains 80 % of its initial capacity after 10 000 cycles at 5 A g**^−1^**, balancing rate capability with cycle stability. Although not every single metric is record-breaking, the simultaneous optimisation of operating voltage, energy density and long-term durability positions this electrode as one of the most competitive options yet reported for high-performance quasi-solid-state zinc-ion energy-storage devices.

**Table S6.** Comparison of electrochemical properties of different vanadium oxides composites and carbon materials applied to quasi-solid-state ZIBs (zinc foil as anode)

| **Cathode** | **Voltage Window (V)** | **Energy density (Wh kg^−1^)** | **Current density**  **(A g^−1^)/Cycle num**b**er/Capacity retention** | **Electrolyte** |
| --- | --- | --- | --- | --- |
| **C@V_2_O_3_ aerogel**  **(This work)** | **0-2** | **415** | **5/10000/80%** | **gelatin/ ZnSO_4_** |
| VO_2_@C ^[44]^ | 0.2-1.6 |  | 3/1000/67% | PAM/ZnSO_4_ |
| V_2_O_5_@CNTs ^[28]^ | 0.2-1.6 | 91.27 | 5.6(mA/cm^2^)/300/90% | PVA/Zn(CF_3_SO_3_)_2_ |
| NVO ^[45]^ | 0.4-1.4 | 214 | 3/750/69% | PAM/ZnSO_4_ |
| MnO_2_/CNT ^[46]^ | 1-1.8 | 364 | 1C/150/90% | xanthan gum |
| NPFCs ^[47]^ | 0.1-1.8 | 60.1 | 5/10000/99% | PAM/Zn(CF_3_SO_3_)_2_ |
| LDC ^[48]^ | 0.2-1.8 | 86.8 | 5/6500/81.3% | gelatin/ZnSO_4_ |
| TAP/Ti_3_C_2_T_x_ ^[43]^ | 0.2-1.5 |  | 1/1000/80.4% | PAM/ZnSO_4_ |
| RbPC ^[49]^ | 0.2-1.8 | 163.2 | 10/20000/98.8% | gelatin/Zn(CF_3_SO_3_)_2_ |

**References**

1. H. Chen, N. Sun, Q. Zhu, R. A. Soomro, and B. Xu, “Microcrystalline Hybridization Enhanced Coal‐Based Carbon Anode for Advanced Sodium‐Ion Batteries,” Advanced Science 9, no. 20 (2022): 2200023.

https://doi.org/10.1002/advs.202200023

2. J. Liao, Y. Zhang, and L. Zhang, “Improved Catalytic Activity on the Thermal Decomposition of Ammonium Perchlorate and Efficient Adsorption of Uranium Using a Novel Ultra-Low Density Al_2_O_3_-Based Aerogels,” Journal of Hazardous Materials 387, (2020): 122015. https://doi.org/10.1016/j.jhazmat.2020.122015

3. Y. Chen, G. Mao, Y. Tang, et al., “Synthesis of Core-Shell Nanostructured Cr_2_O_3_/C@TiO_2_ for Photocatalytic Hydrogen Production,” Chinese Journal of Catalysis 42, no. 1 (2021): 225. https://doi.org/10.1016/s1872-2067(20)63615-4

4. C. Park, E. Samuel, B. Joshi, et al., “Supersonically Sprayed Fe_2_O_3_/C/CNT Composites for Highly Stable Li-Ion Battery Anodes,” Chemical Engineering Journal 395, (2020): 125018. https://doi.org/10.1016/j.cej.2020.125018

5. K. W. Zhu, S., H. Shou, F. Shen, et al., “Defect Engineering on V_2_O_3_ Cathode for Long-Cycling Aqueous Zinc Metal Batteries,” Nature Communications 12, no. 1 (2021): 6878. https://doi.org/10.1038/s41467-021-27203-w

6. J. Duan, K. Ji, L. Min, et al., “Hybrid Electrolyte-Mediated Nano-Scaled γ-Fe_2_O_3_ Cathode for Emerging Aqueous Zinc Battery,” Electrochimica Acta 390, (2021): 138883. https://doi.org/10.1016/j.electacta.2021.138883

7. X. Chen, Q. Kong, X. Wu, et al., “V_2_O_3_@C Optimized by Carbon Regulation Strategy for Ultra Long-Life Aqueous Zinc-Ion Batteries,” Chemical Engineering Journal 451, (2023): 138765. https://doi.org/10.1016/j.cej.2022.138765

8. T. Zhang, L. Zhang, L. Zhao, et al., “Free-Standing, Foldable V_2_O_3_/Multichannel Carbon Nanofibers Electrode for Flexible Li-Ion Batteries with Ultralong Lifespan,” Small 16, no. 47 (2020): e2005302. https://doi.org/10.1002/smll.202005302

9. J. Wang, G. Li, X. Liu, et al., “In-Situ Electrochemical Oxidization of V_2_O_3_-C Cathode for Boosted Zinc-Ion Storage Performance,” Applied Surface Science 616, no. (2023): 156481. https://doi.org/10.1016/j.apsusc.2023.156481

10. H. Lu, Z. Zhang, X. An, J. Feng, S. Xiong, and B. Xi, “In Situ Electrochemically Transforming VN/V_2_O_3_ Heterostructure to Highly Reversible V_2_NO for Excellent Zinc Ion Storage,” Small Structures 4, no. 11 (2023): 2300191.

https://doi.org/10.1002/sstr.202300191

11. R. Wang, W. Wang, M. Sun, Y. Hu, and G. Wang, “Long‐lifespan Zinc‐ion Capacitors Enabled by Anodes Integrated with Interconnected Mesoporous Chitosan Membranes through Electrophoresis‐driven Phase Separation,” Angewandte Chemie International Edition 63, no. 10 (2024): e202317154.

https://doi.org/10.1002/anie.202317154

12. H. Liang, B. Hai, Y. Wang, et al., “Designing Layered V_2_O_3_@C with Stable Oxygen Defects via UV-Curing Technology for High-Performance Zn-Ion Hybrid Supercapacitor,” Applied Surface Science 622, (2023): 156951.

https://doi.org/10.1016/j.apsusc.2023.156951

13. J. Zheng, Y. Zhang, C. Meng, et al., “V_2_O_3_/C Nanocomposites with Interface Defects for Enhanced Intercalation Pseudocapacitance,” Electrochimica Acta 318, (2019): 635. https://doi.org/10.1016/j.electacta.2019.06.125

14. H. Wu, Z. Zhang, M. Qin, et al., “Solution Combustion Synthesis of Crystalline V_2_O_3_ and Amorphous V_2_O_3_/C as Anode for Lithium-Ion Battery,” Journal of the American Ceramic Society 103, no. 4 (2019): 2643. https://doi.org/10.1111/jace.16962

15. J.-H. Kim, Y.-S. Kim, S.-H. Moon, et al., “Enhanced Electrochemical Performance of a Selectively Formed V_2_O_3_/C Composite Structure for Li-Ion Batteries,” Electrochimica Acta 389, (2021): 138685.

https://doi.org/10.1016/j.electacta.2021.138685

16. U. Jančič, M. Božič, S. Hribernik, T. Mohan, R. Kargl, K. S. Kleinschek, et al., “High Oxygen Barrier Chitosan Films Neutralized by Alkaline Nanoparticles,” Cellulose 28, no. 16 (2021): 10457–10475. <https://doi.org/10.1007/s10570-021-04195-w>

17. C. S. Liu, X. Ye, B. Zhou, et al., “2D Mesoporous Nanomesh from N-Doped Carbon-Encapsulated V_2_O_3_ Nanowires as an Anode for Lithium-Ion Batteries,” The Journal of Physical Chemistry C 124, no. 44 (2020): 24073.

https://doi.org/10.1021/acs.jpcc.0c06899

18. P. Liang, K. Zhu, Y. Rao, et al., “Flexible and Self-Standing Urchinlike V_2_O_3_@Carbon Nanofibers toward Ultralong Cycle Lifespan Lithium-Ion Batteries,” ACS Applied Energy Materials 5, no. 3 (2022): 3242.

https://doi.org/10.1021/acsaem.1c03854

19. X. Liu, Z. Wang, Y. Niu, et al., “Electrospun V_2_O_3_@Carbon Nanofibers as a Flexible and Binder-Free Cathode for Highly Stable Aqueous Zn-Ion Full Batteries,” ACS Applied Energy Materials 5, no. 3 (2022): 3525.

https://doi.org/10.1021/acsaem.1c04068

20. Y. Ding, Y. Peng, S. Chen, et al., “Hierarchical Porous Metallic V_2_O_3_@C for Advanced Aqueous Zinc-Ion Batteries,” ACS Applied Materials & Interfaces 11, no. 47 (2019): 44109. https://doi.org/10.1021/acsami.9b13729

21. D. Wang, W. Liang, X. He, et al., “V_2_O_3_@C Microspheres as the High-Performance Cathode Materials for Advanced Aqueous Zinc-Ion Storage,” ACS Applied Materials & Interfaces 15, no. 17 (2023): 20876.

https://doi.org/10.1021/acsami.2c21763

22. H. Shi, J. Cao, W. Sun, et al., “Ultrasmall, Amorphous V_2_O_3_ Intimately Anchored on a Carbon Nanofiber Aerogel Toward High-Rate Zinc-Ion Batteries,” ACS Applied Materials & Interfaces 16, (2024): 18812–18823.

https://doi.org/10.1021/acsami.3c19533

23. Y. Niu, D. Wang, Y. Ma, and L. Zhi, “Cascading V_2_O_3_/N-doped Carbon Hybrid Nanosheets as High-Performance Cathode Materials for Aqueous Zinc-Ion Batteries,” Chinese Chemical Letters 33, no. 3 (2022): 1430.

https://doi.org/10.1016/j.cclet.2021.08.058

24. X. Wang, Z. Zhang, M. Huang, J. Feng, S. Xiong, and B. Xi, “In Situ Electrochemically Activated Vanadium Oxide Cathode for Advanced Aqueous Zn-Ion Batteries,” Nano Letters 22, no. 1 (2021): 119.

https://doi.org/10.1021/acs.nanolett.1c03409

25. T.-T. Lv, X. Luo, G.-Q. Yuan, S.-Y. Yang, and H. Pang, “Layered VO_2_@N-doped Carbon Composites for High-Performance Rechargeable Aqueous Zinc-Ion Batteries,” Chemical Engineering Journal 428, (2022): 131211.

https://doi.org/10.1016/j.cej.2021.131211

26. M. Yang, D. Ma, H. Mi, et al., “A Unique Morphology and Interface Dual-Engineering Strategy Enables the Holey C@VO_2_ Cathode with Enhanced Storage Kinetics for Aqueous Zn-Ion Batteries,” Journal of Materials Chemistry A 9, no. 13 (2021): 8792. https://doi.org/10.1039/d1ta00892g

27. C. Liu, R. Li, W. Liu, G. Shen, and D. Chen, “Chitosan-Assisted Fabrication of a Network C@V_2_O_5_ Cathode for High-Performance Zn-Ion Batteries,” ACS Appl. Mater. Interfaces 13, no. 31 (2021): 37194. https://doi.org/10.1021/acsami.1c09951

28. X. Wang, Y. Wang, J. Hao, et al., “Pseudocapacitive Zinc Cation Intercalation with Superior Kinetics Enabled by Atomically Thin V_2_O_5_ Nanobelts for Quasi-Solid-State Microbatteries,” Energy Storage Materials 50, (2022): 454.

https://doi.org/10.1016/j.ensm.2022.05.049

29. H. Jiang, Y. Zhang, L. Xu, et al., “Fabrication of (NH_4_)_2_V_3_O_8_ Nanoparticles Encapsulated in Amorphous Carbon for High Capacity Electrodes in Aqueous Zinc Ion Batteries,” Chemical Engineering Journal 382, (2020): 122844.

https://doi.org/10.1016/j.cej.2019.122844

30. P. Hu, T. Zhu, X. Wang, et al., “Aqueous Zn//Zn(CF_3_SO_3_)_2_//Na_3_V_2_(PO_4_)_3_ Batteries with Simultaneous Zn^2+^/Na^+^ Intercalation/De-Intercalation,” Nano Energy 58, (2019): 492. https://doi.org/10.1016/j.nanoen.2019.01.068

31. J. Ding, H. Zheng, H. Gao, et al., “In Situ Lattice Tunnel Distortion of Vanadium Trioxide for Enhancing Zinc Ion Storage,” Advanced Energy Materials 11, no. 26 (2021): 2100973. https://doi.org/10.1002/aenm.202100973

32. J. Ding, H. Gao, K. Zhao, et al., “In-Situ Electrochemical Conversion of Vanadium Dioxide for Enhanced Zinc-Ion Storage with Large Voltage Range,” Journal of Power Sources 487, (2021): 229369. https://doi.org/10.1016/j.jpowsour.2020.229369

33. X. Chen, L. Wang, H. Li, F. Cheng, and J. Chen, “Porous V_2_O_5_ Nanofibers as Cathode Materials for Rechargeable Aqueous Zinc-Ion Batteries,” Journal of Energy Chemistry 38, (2019): 20. https://doi.org/10.1016/j.jechem.2018.12.023

34. D. Kundu, S. Hosseini Vajargah, L. Wan, B. Adams, D. Prendergast, and L. F. Nazar, “Aqueous vs. Nonaqueous Zn-Ion Batteries: Consequences of the Desolvation Penalty at the Interface,” Energy & Environmental Science 11, no. 4 (2018): 881.

https://doi.org/10.1039/c8ee00378e

35. Y. Yang, Y. Tang, S. Liang, et al., “Transition Metal Ion-Preintercalated V_2_O_5_ as High-Performance Aqueous Zinc-Ion Battery Cathode with Broad Temperature Adaptability,” Nano Energy 61, (2019): 617.

https://doi.org/10.1016/j.nanoen.2019.05.005

36. N. Liu, X. Wu, L. Fan, et al., “Intercalation Pseudocapacitive Zn^2+^ Storage with Hydrated Vanadium Dioxide toward Ultrahigh Rate Performance,” Advanced Materials 32, no. 42 (2020): 1908420. https://doi.org/10.1002/adma.201908420

37. L. Dong, X. Ma, Y. Li, et al., “Extremely Safe, High-Rate and Ultralong-Life Zinc-Ion Hybrid Supercapacitors,” Energy Storage Materials 13, (2018): 96.

https://doi.org/10.1016/j.ensm.2018.01.003

38. P. Liu, W. Liu, Y. Huang, P. Li, J. Yan, and K. Liu, “Mesoporous Hollow Carbon Spheres Boosted, Integrated High-Performance Aqueous Zn-Ion Energy Storage,” Energy Storage Materials 25, (2020): 858. https://doi.org/10.1016/j.ensm.2019.09.004

39. R. Fei, H. Wang, Q. Wang, et al., “In Situ Hard‐Template Synthesis of Hollow Bowl‐Like Carbon: A Potential Versatile Platform for Sodium and Zinc Ion Capacitors,” Advanced Energy Materials 10, no. 47 (2020): 2002741.

https://doi.org/10.1002/aenm.202002741

40. F. Wei, H. Zhang, X. Hui, Y. Lv, S. Ran, and X. Liu, “N-Doped Porous Carbon Nanosheets with Enhanced Zinc Ion Storage Capability,” Journal of Power Sources 554, (2023): 232348. https://doi.org/10.1016/j.jpowsour.2022.232348

41. H. Zhang, Z. Chen, Y. Zhang, et al., “Boosting Zn-Ion Adsorption in Cross-Linked N/P Co-Incorporated Porous Carbon Nanosheets for the Zinc-Ion Hybrid Capacitor,” Journal of Materials Chemistry A 9, no. 30 (2021): 16565.

https://doi.org/10.1039/d1ta03501k

42. Y. Zhu, X. Ye, H. Jiang, et al., “Controlled Swelling of Graphene Films Towards Hierarchical Structures for Supercapacitor Electrodes,” Journal of Power Sources 453, (2020): 227851. https://doi.org/10.1016/j.jpowsour.2020.227851

43. X. Wang, Y. Liu, Z. Wei, et al., “MXene‐Boosted Imine Cathodes with Extended Conjugated Structure for Aqueous Zinc‐Ion Batteries,” Advanced Materials 34, no. 50 (2022): 2206812. https://doi.org/10.1002/adma.202206812

44. Y. Liu, Y. Liu, X. Wu, and Y.-R. Cho, “Enhanced Electrochemical Performance of Zn/VO_x_ Batteries by a Carbon-Encapsulation Strategy,” ACS Applied Materials & Interfaces 14, no. 9 (2022): 11654. https://doi.org/10.1021/acsami.2c00001

45. Y. Zheng, C. Tian, Y. Wu, et al., “Dual-Engineering of Ammonium Vanadate for Enhanced Aqueous and Quasi-Solid-State Zinc Ion Batteries,” Energy Storage Materials 52, (2022): 664. https://doi.org/10.1016/j.ensm.2022.08.036

46. S. Zhang, N. Yu, S. Zeng, et al., “An Adaptive and Stable Bio-Electrolyte for Rechargeable Zn-Ion Batteries,” Journal of Materials Chemistry A 6, no. 26 (2018): 12237. https://doi.org/10.1039/c8ta04298e

47. F. Wei, Y. Wei, J. Wang, M. Han, and Y. Lv, “N, P Dual-Doped Foamy-Like Carbons with Abundant Defect Sites for Zinc Ion Hybrid Capacitors,” Chemical Engineering Journal 450, (2022): 137919. https://doi.org/10.1016/j.cej.2022.137919

48. Y. Lu, Z. Li, Z. Bai, et al., “High Energy-Power Zn-Ion Hybrid Supercapacitors Enabled by Layered B/N Co-Doped Carbon Cathode,” Nano Energy 66, (2019): 104132. https://doi.org/10.1016/j.nanoen.2019.104132

49. L. Wang, M. Peng, J. Chen, et al., “High Energy and Power Zinc Ion Capacitors: A Dual-Ion Adsorption and Reversible Chemical Adsorption Coupling Mechanism,” ACS Nano 16, no. 2 (2022): 2877. https://doi.org/10.1021/acsnano.1c09936
